# Logistic random effects regression models: a comparison of statistical packages for binary and ordinal outcomes

**DOI:** 10.1186/1471-2288-11-77

**Published:** 2011-05-23

**Authors:** Baoyue Li, Hester F Lingsma, Ewout W Steyerberg, Emmanuel Lesaffre

**Affiliations:** 1 Department of Biostatistics, Erasmus MC, Dr. Molewaterplein 50, Rotterdam, the Netherlands; 2 Department of Public Health, Erasmus MC, Dr. Molewaterplein 50, Rotterdam, the Netherlands; 3 L-Biostat, K.U.Leuven, Kapucijnenvoer 35, Leuven, Belgium

## Abstract

**Background:**

Logistic random effects models are a popular tool to analyze multilevel also called hierarchical data with a binary or ordinal outcome. Here, we aim to compare different statistical software implementations of these models.

**Methods:**

We used individual patient data from 8509 patients in 231 centers with moderate and severe Traumatic Brain Injury (TBI) enrolled in eight Randomized Controlled Trials (RCTs) and three observational studies. We fitted logistic random effects regression models with the 5-point Glasgow Outcome Scale (GOS) as outcome, both dichotomized as well as ordinal, with center and/or trial as random effects, and as covariates age, motor score, pupil reactivity or trial. We then compared the implementations of frequentist and Bayesian methods to estimate the fixed and random effects. Frequentist approaches included R (lme4), Stata (GLLAMM), SAS (GLIMMIX and NLMIXED), MLwiN ([R]IGLS) and MIXOR, Bayesian approaches included WinBUGS, MLwiN (MCMC), R package MCMCglmm and SAS experimental procedure MCMC.

Three data sets (the full data set and two sub-datasets) were analysed using basically two logistic random effects models with either one random effect for the center or two random effects for center and trial. For the ordinal outcome in the full data set also a proportional odds model with a random center effect was fitted.

**Results:**

The packages gave similar parameter estimates for both the fixed and random effects and for the binary (and ordinal) models for the main study and when based on a relatively large number of level-1 (patient level) data compared to the number of level-2 (hospital level) data. However, when based on relatively sparse data set, i.e. when the numbers of level-1 and level-2 data units were about the same, the frequentist and Bayesian approaches showed somewhat different results. The software implementations differ considerably in flexibility, computation time, and usability. There are also differences in the availability of additional tools for model evaluation, such as diagnostic plots. The experimental SAS (version 9.2) procedure MCMC appeared to be inefficient.

**Conclusions:**

On relatively large data sets, the different software implementations of logistic random effects regression models produced similar results. Thus, for a large data set there seems to be no explicit preference (of course if there is no preference from a philosophical point of view) for either a frequentist or Bayesian approach (if based on vague priors). The choice for a particular implementation may largely depend on the desired flexibility, and the usability of the package. For small data sets the random effects variances are difficult to estimate. In the frequentist approaches the MLE of this variance was often estimated zero with a standard error that is either zero or could not be determined, while for Bayesian methods the estimates could depend on the chosen "non-informative" prior of the variance parameter. The starting value for the variance parameter may be also critical for the convergence of the Markov chain.

## Background

Hierarchical, multilevel, or clustered data structures are often seen in medical, psychological and social research. Examples are: (1) individuals in households and households nested in geographical areas, (2) surfaces on teeth, teeth within mouths, (3) children in classes, classes in schools, (4) multicenter clinical trials, in which individuals are treated in centers, (5) meta-analyses with individuals nested in studies. Multilevel data structures also arise in longitudinal studies where measurements are clustered within individuals.

The multilevel structure induces correlation among observations within a cluster, e.g. between patients from the same center. An approach to analyze clustered data is the use of a multilevel or random effects regression analysis. There are several reasons to prefer a random effects model over a traditional fixed effects regression model [1]. First, we may wish to estimate the effect of covariates at the group level, e.g. type of center (university versus peripheral center). With a fixed effects model it is not possible to separate out group effects from the effect of covariates at the group level. Secondly, random effects models treat the groups as a random sample from a population of groups. Using a fixed effects model, inferences cannot be made beyond the groups in the sample. Thirdly, statistical inference may be wrong. Indeed, traditional regression techniques do not recognize the multilevel structure and will cause the standard errors of regression coefficients to be wrongly estimated, leading to an overstatement or understatement of statistical significance for the coefficients of both the higher- and lower-level covariates.

All this is common knowledge in the statistical literature [2], but in the medical literature still multilevel data are often analyzed using fixed effects models [3].

In this paper we use a multilevel dataset with an ordinal outcome, which we analysed as such but also in a dichotomized manner as a binary outcome. Relating patient and cluster characteristics to the outcome requires some special techniques like a logistic (or probit, cloglog, etc) random effects model. Such models are implemented in many different statistical packages, all with different features and using different computational approaches. Packages that use the same numerical techniques are expected to yield the same results, but results can differ if different numerical techniques are used. In this study we aim to compare different statistical software implementations, with regard to estimation results, their usability, flexibility and computing time. The implementations include both frequentist and Bayesian approaches. Statistical software for hierarchical models has been compared already by Zhou et al [4], Guo et al [5] about ten years ago, and by the Centre for Multilevel Modelling (CMM) website [6]. Our paper is different from previous reviews in that we have concentrated on partly different packages and on more commonly used numerical techniques nowadays. Moreover, we considered a binary as well as an ordinal outcome.

## Methods

### Data

The dataset we used here is the IMPACT (International Mission on Prognosis and Clinical Trial design in TBI) database. This dataset contains individual patient data from 9,205 patients with moderate and severe Traumatic Brain Injury (TBI) enrolled in eight Randomized Controlled Trials (RCTs) and three observational studies. The patients were treated in different centers, giving the data a multilevel structure. For more details on this study, we refer to Marmarou et al [7], and Maas et al [8]. The permission to access the patient data used in this study was obtained from the principle investigators of the original studies.

The outcome in our analyses is the Glasgow Outcome Scale (GOS), the commonly used outcome scale in TBI studies. GOS has an ordinal five point scale, with categories respectively dead, vegetative state, severe disability, moderate disability and good recovery. We analyzed GOS on the original ordinal scale but also as a binary outcome, dichotomized into "unfavourable" (dead, vegetative and severe disability) versus "favourable" (good recovery and moderate disability).

At patient level, we included age, pupil reactivity and motor score at admission as predictors in the model, their inclusion is motivated by previous studies [9]. Age was treated as a continuous variable. Motor score and pupil reactivity were treated as categorical variables (motor score: 1 = none or extension, 2 = abnormal flexion, 3 = normal flexion, 4 = localises or obeys, 5 = untestable, and pupil reactivity: 1 = both sides positive, 2 = one side positive, 3 = both sides negative). Note that treatment was not included in our analysis because of absence of a treatment effect in any of the trials. For further details, see McHugh et al [10].

We did include the variable trial since 11 studies were involved and the overall outcome may vary across studies. The trial effect was modelled as a fixed effect in the first analyses and as a random effect in the subsequent analyses. The 231 centers were treated as a random effect (random intercept).

Two sub-datasets were generated in order to examine the performance of the software packages when dealing with logistic random effects regression models on a smaller data set. Sample 1 (cases 2 and 5) consists of a simple random sample from the full data set and contains 500 patients. Sample 2 (cases 3 and 6) was obtained from stratified random sampling the full data set with the centers as strata. It includes 262 patients, representing about 3% of the patients in each hospital.

### Random effects models

In random effects models, the residual variance is split up into components that pertain to the different levels in the data [11]. A two-level model with grouping of patients within centers would include residuals at the patient and center levels. Thus the residual variance is partitioned into a between-center component (the variance of the center-level residuals) and a within-center component (the variance of the patient-level residuals). The center residuals, often called "center effects", represent unobserved center characteristics that affect patients' outcomes. For the cross-classified random effects model (cases 4-6, see below for a description of the model), data are cross-classified by trial and center because some trials were conducted in more than one center and some centers were involved in more than one trial. Therefore, both trial and center were taken as random effects such that the residual variance is partitioned into three parts: a between-trial component, a between-center component and the residual. Note that for the logistic random effects model the level-1 variance is not identifiable from the likelihood; the classically reported fixed variance of pertains to the latent continuous scale and is the variance of *π*^2^/3 a standard logistic density, see Snijders et al [12] and Rodriguez et al [13].

#### Case 1: logistic random effects model on full data set

A dichotomous or binary logistic random effects model has a binary outcome (Y = 0 or 1) and regresses the log odds of the outcome probability on various predictors to estimate the probability that Y = 1 happens, given the random effects. The simplest dichotomous 2-level model is given by(1.1)

with *Y_ij _*the dichotomized GOS (with *Y_ij _*= 1 if GOS = 1,2,3 and *Y_ij _*= 0 otherwise) of the *i *th subject in the *j *th center. Further, *x_ij _*= (*x*_1*ij*_,...,*x_kij _*represents the (first and second level) covariates, *α*_1 _is the intercept and *β_k _*is the *k *th regression coefficient. Furthermore, *u_j _*is the random effect representing the effect of the *j *th center. It is assumed that *u_j _*follows a normal distribution with mean 0 and variance *σ*^2^. Here *x_kij _*represents the covariates age, motor score, pupil reactivity and trial. The coefficient *β_k _*measures the effect of increasing *x_kij _*by one unit on the log odds ratio.

For an ordinal logistic multilevel model, we adopt the proportional odds assumption and hence we assume that:(1.2)

In model (1.2), *Y_ij _*is the GOS of the *i *th subject in the *j *th center. This equation can be seen as a combination of 4 sub-equations. The difference of the four sub-equations is only in the intercept, and the effect of the covariates is assumed to be the same for all outcome levels (proportional odds assumption). So the coefficient *β_k _*is the log odds ratio of a higher GOS versus a lower GOS when the predictor *x_kij _*increases with one unit controlling for the other predictors and the random effect in the model.

In our basic models we assumed a logit link function and a normal distribution for both the binary and the ordinal analysis, but we checked also whether different link functions and other random effect distributions are available in the packages.

#### Cases 2 and 3

Case 2 is based on sample 1 (500 patients), while case 3 is based on sample 2 (262 patients). For both cases only the binary logistic random effects model (1.1) was fitted to the data.

#### Case 4: cross-classified logistic random effects model on full data set

For this case we treated trial (describing 11 studies) as a second random effect. Since trial is not nested in center, we obtained the following cross-classified random effects model:(1.3)

with *Y_ij1 _*is the GOS of the *i *th subject in the *j *th center and the *l *th trial, and *x_ij _*= (*x*_1*ij*1_,...,*x_kijL_*). Note that equations (1.3) and (1.1) differ only in the additional part *v_l _*which represents the random effect of the *l *th trial. We assumed that both random effects are independently normally distributed.

#### Cases 5 and 6

Case 5 is based on sample 1 and case 6 on sample 2. For both cases model (1.3) was fitted to the data.

For more background on models for hierarchical (clustered) data and also for other types of models, such as marginal Generalized Estimating Equations models the reader is referred to the review of Pendergast et al [14].

### Software packages

We compared ten different implementations of logistic random effects models. The software packages can be classified according to the statistical approach upon which they are based, i.e.: frequentist or Bayesian. See Additional file 1 for the different philosophy upon which frequentist and Bayesian approaches are based. We first note that both approaches involve the computation of the likelihood or quasi-likelihood. In the frequentist approach parameter estimation is based on the marginal likelihood obtained from expression (1.2) and (1.3) by integrating out the random effects. In the Bayesian approach all parameters are estimated via MCMC sampling methods.

The frequentist approach is included in the R package lme4, in the GLLAMM package of Stata, in the SAS procedures GLIMMIX and NLMIXED (SAS version 9.2), in the package MLwiN ([R]IGLS version 2.13) and in the program MIXOR (the first program launched for the analysis of a logistic random effects model).

The frequentist approaches differ mainly in the way the integrated likelihood is computed in order to obtain the parameter estimates called maximum likelihood estimate (MLE) or restricted maximum likelihood estimate (REML) depending on the way the variances are estimated. Performing the integration is computationally demanding, especially in the presence of multivariate random effects. As a result, many approximation methods have been suggested to compute the integrated (also called marginal) likelihood. The R package lme4 is based on the Laplace technique, which is the simplest Adaptive Gaussian Quadrature (AGQ) technique based on the evaluation of the function in a well chosen quadrature point per random effect. In the general case, AGQ is a numerical approximation to the integral over the whole support of the likelihood using Q quadrature points adapted to the data [15]. We used the "adapt" option in GLLAMM in Stata to specify the AGQ method [16]. The SAS procedure GLIMMIX allows for several integration approaches and we used AGQ if available [17]. The same holds for the SAS procedure NLMIXED [18]. The package MLwiN ([R]IGLS) adopts Marginal Quasi-Likelihood (MQL) or Penalised quasi-Likelihood (PQL) to achieve the approximation. Both methods can be computed up to the 2^nd ^order [19], here we chose the 2^nd ^order PQL procedure. Finally, in MIXOR, only Gauss-Hermite quadrature, also known as a non-AGQ method, is available. Again the number of quadrature points Q determines the desired accuracy [20]. However Lesaffre and Spiessens indicated that this method can give a poor approximation to the integrated likelihood when the number of quadrature points is low (say 5, which is the default in MIXOR) [21]. Therefore in our analyses we have taken 50 quadrature points but we also applied MIXOR with 5 quadrature points to indicate the sensitivity of the estimation procedure to the choice of Q.

With regard to the optimization technique to obtain the (R)MLE, a variety of techniques are available. R package lme4 uses the NLMINB method which is a local minimiser for the smooth nonlinear function subject to bound-constrained parameters. Newton-Raphson is the only optimization technique in the GLLAMM package. SAS procedures GLIMMIX and NLMIXED have a large number of optimization techniques. We chose the default Quasi-Newton approach for GLIMMIX and the Newton-Raphson algorithm for NLMIXED. The package MLwiN ([R]IGLS) adopts iterative generalised least squares (IGLS) or restricted IGLS (RIGLS) optimization methods. We used IGLS although it has been shown that RIGLS yields less biased estimates than IGLS [22], we will return to this below. Finally, in MIXOR, the Fisher-scoring algorithm was used.

It has been documented that quasi-likelihood approximations such as those implemented in MLwiN ([R]IGLS) may produce estimates biased towards zero in certain circumstances. The bias could be substantial especially when data are sparse [23,24]. On the other hand, (adaptive) quadrature methods with an adequate number of quadrature points produce less biased estimates [25].

Note that certain integration and optimization techniques are not available in some software for a cross-classified logistic random effects model. This will be discussed later.

The other four programs we studied are based on a Bayesian approach. The program most often used for Bayesian analysis is WinBUGS (latest and final version is 1.4.3). WinBUGS is based on the Gibbs Sampler, which is one of the MCMC methods [26]. The package MLwiN (using MCMC) allows for a multilevel Bayesian analysis, it is based on a combination of Gibbs sampling and Metropolis-Hastings sampling [27], both examples of MCMC sampling. The R package MCMCglmm is designed for fitting generalised linear mixed models and makes use of MCMC techniques that are a combination of Gibbs sampling, slice sampling and Metropolis-Hastings sampling [28]. Finally, the recent experimental SAS 9.2 procedure MCMC is a general purpose Markov Chain Monte Carlo simulation procedure that is designed to fit many Bayesian models using the Metropolis-Hastings approach [29].

In all Bayesian packages we used "non-informative" priors for all the regression coefficients, i.e. a normal distribution with zero mean and a large variance (10^4^). Note that, the adjective "non-informative" prior used in this paper is the classical wording but does not necessarily mean the prior is truly non-informative, as will be seen below. The random effect is assumed to follow a normal distribution and the standard deviation of the random effects is given a uniform prior distribution between 0 and 100. MLwiN, however, uses the Inverse Gamma distribution for the variance as default. Since the choice of the non-informative prior for the standard deviation can seriously affect the estimation of all parameters, other priors for the standard deviation were also used. The total number of iterations for binary models in all cases (except for cases 3 and 6) was 10,000 with a burn-in of 3,000. More iterations (10^6^) were used in cases 3 and 6 in order to get convergence for the small data set. For the ordinal model in case 1, the total number of iterations was 100,000 and the size of the burn-in part was 30,000.

We checked convergence of the MCMC chain using the Brooks-Gelman-Rubin (BGR) method [30] in WinBUGS. This method compares within-chain and between-chain variability for multiple chains starting at over-dispersed initial values. Convergence of the chain is indicated by a ratio close to 1. In MLwiN (MCMC) the Raftery-Lewis method was used [27]. For MCMCglmm, we used the BGR method by making use of the R-package CODA. The SAS procedure MCMC offers many convergence diagnostic tests, we used the Geweke diagnostic.

The specification of starting values for parameters is a bit different across packages.

Among the six frequentist packages, lme4, NLMIXED and MIXOR allow manual specification of the starting values, while in the other packages default starting values are chosen automatically. NLMIXED uses 1 as starting value for all parameters for which no starting values have been specified. For lme4 and MIXOR the choice of the starting values is not clear, while GLIMMIX and GLLAMM base their default starting values on the estimates from a generalized linear model fit. In MLwiN ([R]IGLS) the 2^nd ^order PQL method uses MQL estimates as starting values. Note that for most Bayesian implementations the starting values should be specified by the user. Often the choices of starting values, if not taken too extreme, do not play a great role in the convergence of the MCMC chain but care needs to be exercised for the variance parameters, as seen below.

### Analysis

As outlined above, binary and ordinal logistic random effects regression models were fitted to the IMPACT data. All packages are able to deal with the binary logistic random effects model. Furthermore, the packages GLLAMM, GLIMMIX, NLMIXED, MLwiN ([R]IGLS), MIXOR, WinBUGS, MLwiN (MCMC) and SAS MCMC are able to analyze ordinal multilevel data. MCMCglmm only supports the probit model for an ordinal outcome, so that program was not used for the ordinal case. The packages R, GLIMMIX, MLwiN ([R]IGLS), WinBUGS, MLwiN (MCMC) and MCMCglmm can handle the cross-classified random effects model. Syntax codes for the analysis of the IMPACT data with the different packages are provided in Additional file 2.

We compared the packages with respect to the estimates of the parameters and the time needed to arrive at the final estimates. Further, we compared extra facilities, output and easy handling of the programs. Finally, we looked at the flexibility of the software, i.e. whether it is possible to vary the model assumptions made in (1.1) and (1.2), e.g. replacing the logit link by other link functions such as probit and log(-log) link functions or relaxing the assumption of normality for the random effects.

## Results

### Descriptive statistics

From the 9,205 patients in the original database, we excluded the patients with a missing GOS at 6 months (n = 484) or when there was only partial information available on GOS (n = 35), or when the age was missing (n = 2) or if the patient was younger than 14 (n = 175). This resulted in 8,509 patients in 231 centers in the analysis, of whom 2,396 (28%) died and 4,082 (48%) had an unfavourable outcome six months after injury (see Additional file 3). The median age was 30 (interquartile range 21-45) years, 3522 patients (41%) had a motor score of 3 or lower (none, extension or abnormal flexion), and 1,989 patients (23%) had bilateral non-reactive pupils. The median number of patients per center was 19, ranging from 1 to 425.

### Case 1: binary and ordinal logistic random effects model on full data set

#### Binary model

Fitting the dichotomous model in the different packages gave similar results (see Additional file 4). For the frequentist approaches the R package lme4, the Stata package GLLAMM, the SAS procedures GLIMMIX and NLMIXED, and the programs MLwiN ([R]IGLS) and MIXOR provided almost the same results for the fixed effects and the variance of the random effects. One example is age, with estimated coefficients of 0.623, 0.623, 0.618, 0.623, 0.623 and 0.623, respectively for the different programs and all estimated SDs close to 0.028. Estimates for the variance of the random effects were also similar: 0.101, 0.102, 0.107, 0.102, 0.101 and 0.102, respectively. As can be noticed from Additional file 4, lme4 did not give an estimate for the SD of the variance of the random effects. The reason was provided by the developer of the package in his book (Bates D: lme4: Mixed-effects modelling with R, submitted) stating that the sampling distribution of the variance is highly skewed which makes the standard error nonsensical.

The Bayesian programs WinBUGS, MLwiN (MCMC), MCMCglmm and the SAS procedure MCMC gave similar posterior means and these were also close to the MLEs obtained from the frequentist software. For example, the posterior mean (SD) of the regression coefficient of age was 0.626 (0.028), 0.625 (0.029), 0.636 (0.028) and 0.630 (0.025) for WinBUGS, MLwiN (MCMC), MCMCglmm and SAS procedure MCMC, respectively. The posterior mean of the variance of the random effects was estimated as 0.119, 0.113, 0.110 and 0.160, respectively with SD close to 0.30.

The random effects estimates of the 231 centers could easily be derived from all packages except for MIXOR and were quite similar. For example the Pearson correlation for the estimated random effects from WinBUGS and R was 0.9999.

#### Ordinal model-proportional odds model

Fitting the proportional odds model in the different packages also gave similar results (see Additional file 5). For the frequentist approach, the Stata package GLLAMM, the two SAS procedures GLIMMIX and NLMIXED, the packages MLwiN ([R]IGLS) and MIXOR gave very similar estimates for the fixed effects parameters and the variance of the random effects. The estimate (SD) of e.g. the regression coefficient of age was 0.591 (0.023), 0.588 (0.023), 0.591(0.023), 0.592 (0.023) and 0.591 (0.027), respectively. The estimate of the variance (SD) of the random effects were 0.085 (0.020), 0.090 (0.021), 0.085 (0.020), 0.085 (0.019), and 0.085 (0.024), respectively. The MIXOR results were somewhat different from those of the other packages when based on 5 quadrature points, but this difference largely disappeared when 50 quadrature points were used, see Additional file 5. However, the SDs did not change much by increasing Q from 5 to 50 and we are not sure about the reason behind.

For the Bayesian approaches, WinBUGS and MLwiN (MCMC) produced similar results as the frequentist approaches. The posterior mean of the regression coefficient of age in WinBUGS was 0.551 and 0.592 in MLwiN (MCMC), with SD = 0.023 in both cases (same as the SAS frequentist result). The posterior mean of the variance of the random effects was 0.096 in WinBUGS and 0.093 in MLwiN (MCMC) and for both SD = 0.022, very close to the frequentist estimates. We stopped running the SAS MCMC procedure after 2,000 iterations because this already took 19 hours and the chains based on the last 1,000 iterations were far from being converged.

Finally, the estimated random effects for the 231 centers were quite the same across the different packages (except for MIXOR) with correlation again practically 1.

### Cases 2 and 3: binary logistic random effects models on samples 1 and 2

The conclusions for case 2 are the same as for case 1 (see Additional file 6), but not for case 3 (see Additional file 7). The results for the Bayesian analyses are rather different from the results of the frequentist implementations but similar to each other, in particular with regard to the posterior standard errors. For the frequentist approaches, the variance of the random effects was estimated zero and the standard error was estimated as zero or could not be estimated. What is more important in case 3 is that the posterior means depended much on the choice of the non-informative priors for the variance component, i.e. uniform (0,1) and Inverse Gamma (0.001,0.001), but we have tried more priors and elaborated on this in the discussion section of the paper.

### Case 4: cross-classified binary logistic random effects model based on full data set

Only lme4 in R, GLIMMIX, MLwiN ([R]IGLS), WinBUGS, MLwiN (MCMC) and MCMCglmm could handle this analysis. The results for these packages were quite similar, as shown in Additional file 8. For example for age the estimates (SD) were 0.623 (0.028), 0.617 (0.028), 0.623 (0.028), 0.624 (0.028), 0.624 (0.027) and 0.635 (0.028), for lme4, GLIMMIX, MLwiN ([R]IGLS), WinBUGS, MLwiN (MCMC) and MCMCglmm, respectively. The variances for the random effect of center were 0.116, 0.113, 0.116, 0.119, 0.120 and 0.106, respectively and for the random effect of trial they were 0.067, 0.075, 0.067, 0.114, 0.095 and 0.094, respectively.

### Cases 5 and 6: cross-classified binary logistic random effects models on samples 1 and 2

As for case 2, we obtained in case 5 essentially the same results with all packages. For case 6, the frequentist results were similar but the Bayesian results were different and were much affected by the prior of the variance parameter as in case 3 (tables for cases 5 and 6 are not shown).

#### Usability, flexibility and speed

The packages greatly differed in their usability, by which we mean the availability of diagnostic tools/plots; ease of displaying/extracting parameter estimates and exporting results, etc. But it must be stated that all packages require a sound statistical knowledge in multilevel modelling in order to analyze such data in a reliable manner.

SAS is based on procedures for which certain options can be turned on and off. Understanding the different options in the statistical SAS procedures often requires a great deal of statistical background since the procedures are based on the most advanced and computationally powerful methods. Also SAS data management is quite powerful but is also associated with a steep learning curve. The SAS procedures NLMIXED and MCMC offer some programming facilities.

The package R has gained a lot of attention in the last decade and is becoming increasingly popular among statisticians and non-statisticians. It requires programming skills and has many basic functions. In addition, R offers great graphics to the user. For the MCMCglmm package in R, we experienced difficulties in understanding the syntax for specifying the prior of the variance parameters as explained in the manual.

Stata is very handy for analyzing simple as well as complicated problems. It has a command-line interface and also includes a graphical user interface since version 8.0. The software allows user-written packages just as in R and provides some programming facilities. The package GLLAMM is powerful in dealing with a large range of complex problems.

WinBUGS is the most popular general purpose package for Bayesian analysis with now more than 30,000 registered users. The package allows for a great variety of analyses using a programming language that resembles to some extent that of R. WinBUGS requires about the same programming skills as R.

MIXOR needs no programming but provides very limited output. Furthermore, MLwiN has a clear and intuitive interface to specify a random effects model, but lacks a simple syntax file structure.

**Figure 1:**
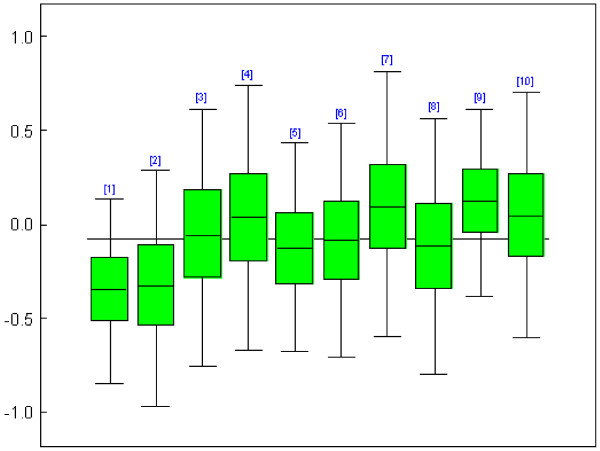
**IMPACT study: Box plot of a sample of the random effects (for center 1 to 10)**. Each box represents a center with its random effects estimate and confidence interval.

**Figure 2:**
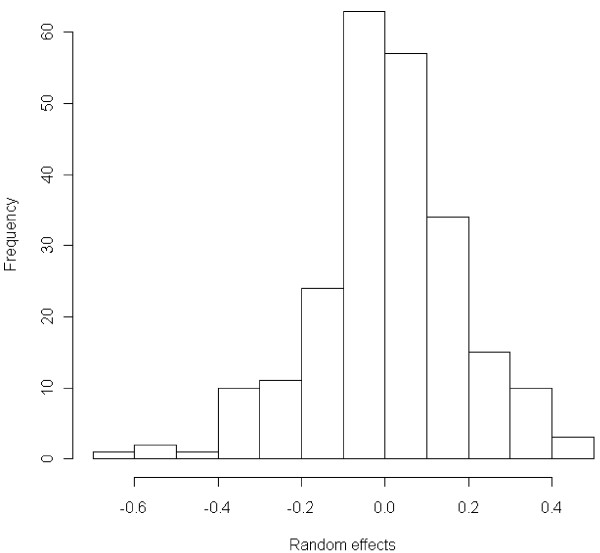
**IMPACT study: Histogram of the random effects in the binary model in R**.

The packages also differ in what they offer as standard output besides the parameter estimates. WinBUGS allows for the most extensive output, including diagnostic plots for model evaluation and plots of the individual random center effects. All packages except MIXOR can provide estimates of the random effects. In Figure 1 we show the box plots of the sampled random effects in WinBUGS for the first 10 centers of the binary logistic random effects model applied to the IMPACT data. Of course with packages like SAS and R the output of the statistical procedures can be saved and then processed by some other procedure or function to deliver the required graph or additional diagnostic analysis. For example, Figure 2 is produced with R and shows the histogram of the random effects of the binary IMPACT logistic random effects model.

Flexibility differs somewhat in the packages. All packages could handle a probit model and a log(-log) model except lme4 and MCMCglmm (MCMCglmm allows for logit or probit link functions for a binary model but only the probit link function for the ordinal model). But, only WinBUGS allows for changing the distribution of the random effects. Table 1 shows that WinBUGS has the greatest flexibility in adapting the model assumptions.

**Table 1:** Extra abilities of different packages

Package	Program/function/option	Link function		Obtaining the random effects	Handling ordinal proportional odds model	Modeling cross-classified model	Other than normal random effects
						
		Probit model	Log (-log) model				
R	LME4			X		X	
	MCMCglmm	X		X	X*	X	

Stata	GLLAMM	X	X	X	X		

MIXOR	MIXOR	X	X		X		

SAS	NLMIXED	X	X	X	X		
	GLIMMIX	X	X	X	X	X	
	MCMC	X	X	X			

MLwiN	[R]IGLS or MCMC	X	X	X	X	X	

WinBUGS	MCMC	X	X	X	X	X	X

*: only probit model is available in MCMCglmm for ordinal model.

The speed of the computations varied widely. All computations were done on an Intel Core(TM) 2 Duo E8400 processor with 3.0 GHz CPU and 3.21 GB internal memory. For case 1, only a few seconds were needed to provide the estimates with the frequentist approaches to fit the binary logistic random effects, except for SAS NLMIXED and Stata GLLAMM which needed 15 minutes and 7 minutes, respectively. The MLwiN ([R]IGLS) procedure (using 2^nd ^order PQL) was the fastest, and GLIMMIX was almost as fast followed by lme4 and MIXOR. The Bayesian approaches were considerably slower, which is not surprising since MCMC sampling is time consuming. However, a major handicap to perform an honest comparison with regard computational speed is that the checking for convergence of MCMC methods is far more difficult than in a frequentist sense [31] and not standardized. Nevertheless, MCMCglmm was the winner this time, but we considered all computation times as acceptable, except for the SAS MCMC procedure which took 37 hours for the binary model. Similar findings were obtained for the ordinal logistic random effects model, but compared to the binary model, the time to converge increased considerably for some software. Now the winner in the frequentist software was GLIMMIX closely followed up by MIXOR. For the Bayesian software, MLwiN (MCMC) was the winner, much faster than WinBUGS. The SAS procedure MCMC never got to convergence (we stopped it) and as mentioned above, the MCMCglmm program does not allow the ordinal logistic random effects model.

## Discussion

### Performance of each package

Although the parameter estimates were very similar between the ten software implementations, we found considerable variations in computing time, usability and flexibility.

#### Speed

Most of the frequentist approaches were very fast, taking only seconds, with the SAS NLMIXED procedure and the Stata package GLLAMM as exceptions. Overall, the SAS procedure GLIMMIX, the program MIXOR and the package MLwiN ([R]IGLS) were the winners. The fact that NLMIXED and GLLAMM took much longer time has much to do with that they are general purpose programs suitable for fitting a variety of complex random effects models and that they both use the AGQ method. The Bayesian approaches were invariably slower than the frequentist approaches, which is due to the computational intensive MCMC approach and that convergence is much harder to judge than in a classical frequentist sense. The speed of the Bayesian procedures appears to depend also more on the sample size than the frequentist approaches. As a result, long processing times as in WinBUGS (14 minutes for binary and 8 hours for ordinal model, respectively) may prevent the user to do much on exploratory statistical research. The R package MCMCglmm and MLwiN (MCMC) were much faster than WinBUGS, taking only a few minutes for both binary and ordinal cases. Hence, from a computational point of view, MCMCglmm and MLwiN (MCMC) are our software of choice for multilevel modeling.

In our experience, the SAS procedure MCMC was inefficient in dealing with mixed models. It was far too time consuming (37 hours for the binary model) and it did converge neither for the regression coefficients nor for the variance of the random effects. At this moment, we cannot recommend this SAS procedure for fitting logistic random effects regression models.

#### Usability and flexibility

The packages differ much in nature, like working interface and data management. MLwiN and MIXOR are menu-driven although writing syntax is also allowed in both packages. SAS is supposed to work in batch mode with some procedures and macros. The others, WinBUGS, R and STATA, are embedded in a programming language. Which package to prefer from the usability viewpoint is difficult to say since it very much depends on the user but also on whether the logistic random effects model fitting is a stand-alone exercise. We know that in practice this is often not the case since we would like to process output of such an analysis to produce e.g. nice graphs. From this viewpoint MIXOR and WinBUGS score lower since they require the user to switch to other software, such as R, to produce additional output or better quality graphs. However, in recent years some packages, like R2WinBUGS in R, can combine WinBUGS and R (or other software) nicely. See the BUGS website [32] to get more information.

For the cross-classified random effects model and the sub-dataset analysis, some integration methods and optimization techniques were not available in some software. For example, in GLIMMIX, AGQ is not available for the cross-classified random effects model and we had to change to Residual Subject-specific Pseudo-Likelihood.

In the R package MCMCglmm, by default the residual variance should be explicitly specified for random effects models. But, as this variance parameter is not identifiable for the logistic random effects model, as seen above, it has to be fixed at a particular value. MCMCglmm uses arbitrary values larger than zero, while the other packages ignore the residual variance since it does not play a role in the estimation process. In order to make the results comparable, the posteriors had to be rescaled which worked most often. But one should be aware that the prior specification will be different after rescaling the posteriors, so there will be differences between MCMCglmm and other Bayesian packages if the prior considerably influences the posterior which happened here for cases 3 and 6.

RIGLS is the restricted version of IGLS in a similar way as REML is a restricted maximum likelihood procedure, with RIGLS less biased especially in linear models, as mentioned before. In logistic random effects models, IGLS was chosen for MLwiN ([R]IGLS) in our study as all other frequentist packages allow for the ML method but not all allow for REML estimation. An additional MLwiN analysis using RIGLS did show somewhat different results. For case 1, the results from RIGLS and IGLS were basically identical, only the variance estimator was 10% higher with RIGLS. For case 3, the regression estimates differed more and the RIGLS estimator of variance was not zero anymore. For more information on ML, REML, etc in different multilevel models, see Browne and Draper [33].

WinBUGS demonstrates much flexibility. Different distributions for the random effects (e.g. gamma, uniform, t-distribution) and different link functions such as probit and log(-log) model are possible. Different link functions are also possible in the SAS procedures GLIMMIX and NLMIXED, but none of these two packages allow for other than normal distributions for the random effects. Note that in our study the binary logistic random effects model was superior to the probit and log(-log) models according to Akaike Information Criterion (using GLIMMIX).

### Problems with small data sets

When the data set is small and the variance of the random effects is near zero, or the ICC (intra-class correlation) is very small as in cases 3 and 6, both frequentist and Bayesian methods can give quite different estimates especially for the variance. The MLE approach might have difficulties estimating small but non-zero variance estimates. The variance was estimated zero with lme4 in R. GLLAMM also estimated the variance as well as its standard error as quite small. GLIMMIX and NLMIXED produced very small estimates for the variance but no output for the standard error. MLwiN ([R]IGLS) estimated the variance and the standard error as zero. Finally, MIXOR gave no output for either the variance or the standard error.

For the Bayesian methods, the posterior means depended much on the choice of prior for the variance component. In order to check their impact, we offered WinBUGS the following three priors for the standard deviation of the random effects: uniform (0,1), uniform (0,10), uniform (0,100), and a uniform (0, 10^6^) as well as an inverse Gamma distribution (0.001,0.001) for the variance. We also offered two priors for the variance in MLwiN (MCMC): inverse Gamma distribution (0.001,0.001) and uniform (0, + ∞). This uniform distribution is actually an improper prior which might lead to an improper posterior. Further, it is not the default choice in MLwiN. However, when the default procedure was taken for the improper uniform prior, i.e. starting values are taken from an initial IGLS run, the starting value for the variance parameter was taken too small and remained so until the MCMC sampling was stopped thereby affecting severely all parameters. For this reason we restarted this MLwiN run with 1 as the starting value which solved this problem. The total number of iterations was 1,100,000 with a burn-in of 100,000 iterations with thinning applied every 10 iterations. Convergence was checked and obtained using the criteria offered in each software package. The results are shown in Additional file 9. We can see that most of the Bayesian estimates are larger than the frequentist MLE from Additional file 7, especially for the variance parameter. The reason is that the posterior distribution is highly skewed for the variance therefore the posterior mean is much larger than the posterior mode whose frequentist counterpart is the MLE. We also notice in Additional file 9 that the variance estimates in MLwiN and WinBUGS using a uniform prior on the variance are greater than the WinBUGS results with uniform priors on the standard deviation, which was mentioned by Gelman [34]. To conclude, for small data sets the choice of the prior matters for the posterior estimates of the parameters, as was also shown by e.g. Spiegelhalter et al [35].

### Comparison with previous studies

Zhou et al (1999) compared 5 packages for generalized linear multilevel models. They compared the estimates, the computing time and the features of the packages. In our study, we compared 10 (popular) packages on similar features. Also Bayesian methods were included. Guo and Zhao (2000) compared statistical software for multilevel modelling of binary data, and they put much emphasis on PQL and MQL. Furthermore, the SAS macro GLIMMIX as well as MLn, the DOS predecessor of MLwiN, were included in their comparison. The latter packages are not in use anymore which makes this comparison now outdated.

The CMM website published an online report (multilevel modelling software reviews) which compared almost 20 packages for the normal linear model, the binary response model, the ordered category model and the cross-classified model [6]. But the packages lme4, MCMCglmm and the SAS procedures GLIMMIX and MCMC were not considered in this review. In addition, we evaluated here also the usability and flexibility of the packages.

## Conclusions

We conclude from our study that for relatively large data sets, the parameter estimates from logistic random effects regression models will probably not be much influenced by the choice of the statistical package. In that case the choice of the statistical implementation should depend on other factors, such as speed and desired flexibility. Based on our study, we conclude that if there is no prior acquaintance with a certain package and preference is given to a frequentist approach, the following packages are to be recommended: MLwiN ([R]IGLS), the R package lme4 and the SAS procedure GLIMMIX. For a Bayesian implementation, we would recommend MLwiN (MCMC) because of its efficiency. If the user is also interested in (perhaps more complicated) statistical analyses other than multilevel modelling then he/she could choose WinBUGS.

Finally, a cautionary remark is necessary, i.e. a "large data set" can still be sparse and hence "large" should be interpreted with some caution. For example, a large data set with many lowest-level units nested within nearly as many higher-level units will act as a "small" data set when a multilevel model is fit. For such data sets the result of the fitting exercise might very much depend on the chosen approach: frequentist or Bayesian. In case a Bayesian package is chosen, the parameter estimates might be much influenced by the priors for the variance of the random effects. Since some packages offer only a quite restricted set of priors (such as MLwiN) for this parameter, the choice of the Bayesian package may have a large impact on the posterior estimates of all parameters for "small" data sets. Finally, also the performance of a Bayesian analysis might very much depend on the chosen starting value for the variance parameter, e.g. when chosen (close to) zero the MCMC might be stuck around zero for a very long time (which happened with MLwiN) and thus affect severely the convergence of the Markov chain.

## Competing interests

The authors declare that they have no competing interests.

## Authors' contributions

BL and EL participated in the statistical analyses and drafted the manuscript. HFL and EWS participated in the statistical analyses. All authors approved the final manuscript.

## Pre-publication history

The pre-publication history for this paper can be accessed here:

http://www.biomedcentral.com/1471-2288/11/77/prepub

## Supplementary Material

Additional file 1 **Frequentist and Bayesian approaches**. Click here for file

Additional file 2 **Programmes**. Click here for file

Additional file 3 **IMPACT study: Descriptive statistics of the study population**. Click here for file

Additional file 4 **IMPACT study: Results of the binary model in case 1 (full data set)**. * The variance of the random effects with its standard error is given. Click here for file

Additional file 5 **IMPACT study: Results from the ordinal model in case 1 (full data set)**. * The variance of the random effects with its standard error is given Click here for file

Additional file 6 **IMPACT study: Results from the binary model in case 2 (sample 1)**. * The variance of the random effects with its standard error is given Click here for file

Additional file 7 **IMPACT study: Results from the binary model in case 3 (sample 2)**. * The variance of the random effects with its standard error is given Click here for file

Additional file 8 **IMPACT study: Results from the cross-classified model in case 4 (full data set)**. * The variance of the random effects with its standard error is given Click here for file

Additional file 9 **Impact of variance component priors on the posterior means in WinBUGS and MLwiN in case 3 (sample 2)**. * The variance of the random effects with its standard error is given. * The first three uniform distributions in WinBUGS are for the standard deviation of the random effects and the rest four prior distributions are for the variance of the random effect Click here for file
