# Supplementary material for: Logistic random effects regression models: a comparison of statistical packages for binary and ordinal outcomes
Source: BMC Med Res Methodol. 2011 May 23;11:77. doi: 10.1186/1471-2288-11-77 (PMC3112198; doi:10.1186/1471-2288-11-77)
Supplement: Additional file 1 — Frequentist and Bayesian approaches. [file 1471-2288-11-77-S1.DOC]

**Frequentist and Bayesian approaches**

In the frequentist approach, probability is defined as a limiting relative frequency. That is, the probability of an event is the limit of the relative frequency of that event in a large number of studies. Further, in frequentist statistics one estimates the unknown but fixed model parameter θ. The estimate of θ is obtained by maximizing the likelihood. Prediction is done given the estimated θ and the uncertainty of the prediction is based on the sampling property of the estimated value of θ [[[1]](#endnote-2)].

In the Bayesian approach the parameter θ is given a probability distribution which expresses our prior knowledge about that parameter. There is still a true value for the parameter [[[2]](#endnote-3)], but the parameter becomes stochastic because of our uncertainty. The Bayesian paradigm is based on Bayes’ Theorem which combines a prior belief (or probability) with the actual observed data to arrive at an updated posterior probability [[[3]](#endnote-4)]. The Bayesian approach implies the calculation of high dimensional integrals. Because the integrals are high dimensional, the Bayesian approach was for about two centuries impossible to be used for real-life problems [[[4]](#endnote-5)].

As can be deduced from above, the two approaches differ in their numerical approach: the frequentist involving some optimization routines for maximising the likelihood function or other ways to estimate parameters and the Bayesian involving numerical techniques that perform integration. Since 1989 a powerful class of numerical procedures, called Markov Chain Monte Carlo (MCMC) techniques [[[5]](#endnote-6)], were launched which revolutionalized the Bayesian approach. The MCMC approach is based on a sampling approach, i.e. the integral is approximated by Monte-Carlo sampling [[[6]](#endnote-7)]. In fact there are two major classes of MCMC techniques: Gibbs sampling and Metropolis-Hastings sampling.

The Bayesian approach involves a prior distribution on the parameters and a likelihood. The posterior estimates depend on these two components. With the same data (likelihood), the posterior estimates may change heavily if different informative priors are used. On the other hand, if the prior is non-informative (NI), i.e. when it does not bring any (or brings in only little) prior information on the parameter the posterior estimates only depend on the likelihood. For a flat NI prior the posterior mode is equal to the classic maximum likelihood estimate (MLE), while for other NI priors the posterior mode is often equal to the MLE. This happened in our study as we used non-informative prior distributions of all the parameters. This is one of the reasons why the results from frequentist and Bayesian approaches are very similar. However, we should notice that in our study, we used the posterior mean and not the mode and they are different in case the posterior distribution is strongly asymmetric.

Further, it should be realized that logistic random effects models involve integration with both the frequentist and the Bayesian approach. In fact, models (1.1) and (1.2) yield conditional likelihoods, conditional on the values of the random effects. Since the random effects are not known, the marginal likelihood is determined which is the likelihood integrated over the distribution of the random effects. Random effects estimates from frequentist methods are often referred to as Empirical Bayes estimates.

1. . Feller W: An introduction to Probability Theory and its Applications. New York: [^Wiley^](http://www.getcited.org/inst/144227)^; 1957^ [↑](#endnote-ref-2)
2. . Gelman A, Carlin JB, Stern HS, Rubin DB: Chapter 4.4 Frequency evaluations of Bayesian inference. Bayesian data analysis, second edition. Chapman & Hall; 2003 [↑](#endnote-ref-3)
3. . Bernardo JM, Smith AFM: Bayesian Theory. London: Wiley; 1994 [↑](#endnote-ref-4)
4. . Gelman A, Carlin JB, Stern HS, Rubin DB: Bayesian Data Analysis, Second Edition . Chapman & Hall; 2003 [↑](#endnote-ref-5)
5. . Gelfand A, Smith A: Sampling based approaches to calculating marginal densities. J American Statist Assoc 1990, 85:398–409. [↑](#endnote-ref-6)
6. . Ripley B: Stochastic Simulation. New York: Wiley; 1987 [↑](#endnote-ref-7)
