# Supplementary material for: Logistic random effects regression models: a comparison of statistical packages for binary and ordinal outcomes
Source: BMC Med Res Methodol. 2011 May 23;11:77. doi: 10.1186/1471-2288-11-77 (PMC3112198; doi:10.1186/1471-2288-11-77)
Supplement: Additional file 2 — Programmes. [file 1471-2288-11-77-S2.DOC]

**Programmes**

**Variable coding**

| **Variable label** | **variable name** | **coding** |
| --- | --- | --- |
| **Motor score** | **Motor** | **1**='none'  **2**='extension'  **3**='abnormal flexion'  **4**='normal flexion'  **5**='localises'  **6**='obeys command'  **9**='untestable' |
| **Pupil reactivity** | **Pupil** | **1**='both side positive'  **2**='one side positive'  **3**='both side negative' |
| **Unfavorate** | **d_unfav** | **0**='favorable'  **1**='unfavorable' |
| **Glasgow Outcome Scale** | **GOS** | **1**='dead'  **2**='vegetative status'  **3**='severe disability'  **4**='moderate disability'  **5**='good recovery' |
| **Study** | **Trial** | Using dummy variables |

**Binary logistic random effects model**

**SAS procedure nlmixed**

**proc** **nlmixed** data=aa_std tech=newrap qpoints=**5**;

parms beta0=**0**,beta1=**0**, beta2=-**2**, beta3=-**1**,beta4=**1**,beta5=**1**,beta6=**1**,beta7=**1**,

beta8=**1**,beta9=**1**,beta10=**1**,beta11=**1**,beta12=**1**,beta13=**1**,beta14=**1**,beta15=**1**,beta16=**1**,beta17=**1**,beta18=**1**,beta19=**1**,s2b=**1**;

eta=beta0+beta2*pupil2+beta3*pupil3+beta1*age+beta4*motor2+beta5*motor3+beta6*motor4+beta7*motor5+beta8*motor6+beta9*motor9+beta10*trial2+beta11*trial3+beta12*trial4+beta13*trial5+beta14*trial6+beta15*trial7+beta16*trial8+beta17*trial9+beta18*trial10+beta19*trial11+b0;

mu=exp(eta)/(**1**+exp(eta));

model d_unfav ~ binary(mu);

random b0 ~ normal(**0**,s2b) subject=center_num out=bin;

**run**;

**SAS procedure glimmix**

**proc** **glimmix** data=aa_std method=quad(qpoints=**5**);

class center_num;

model d_unfav(event=last)=pupil2-pupil3 age motor2-motor6 motor9 trial2-trial11/dist=binary solution;

random intercept/ subject=center_num;

output out=i1 pred=p resid=r pred(NOBLUP)=p1;

**run**;

**SAS procedure mcmc**

**proc** **mcmc** data=aa_std outpost=postout seed=**332786** nmc=**10000** nbi=**3000**

monitor=(beta0-beta19 s2b);

array delta[**231**];

parms beta0 **0** beta1 **0** beta2 -**2** beta3 -**1** beta4 **1** beta5 **1** beta6 **1** beta7 **1** beta8 **1** beta9 **1** beta10 **1** ;

parms beta11 **1** beta12 **1** beta13 **1** beta14 **1** beta15 **1** beta16 **1** beta17 **1** beta18 **1** beta19 **1** ;

parms s2b **1**;

%***group_parms***(delta,**231**, **20**, **1**);

prior beta:~normal(**0**,var=**10000**);

prior delta:~normal(beta0,var=s2b);

prior s2b~uniform(**0.01**,**100**);

w=beta1*age+beta2*pupil2+beta3*pupil3+beta4*motor2+beta5*motor3+beta6*motor4+beta7*motor5+beta8*motor6+beta9*motor9+beta10*trial2+beta11*trial3+beta12*trial4+beta13*trial5+beta14*trial6+beta15*trial7+beta16*trial8+beta17*trial9+beta18*trial10+beta19*trial11;

pi=logistic(w+delta[center_num]);

model d_unfav ~ binary(pi);

**run**;

**R fuction lmer**

glmer(d_unfav~pupil2+pupil3+age+motor2+motor3+motor4+motor5+motor6+motor9+trial2+trial3+trial4+trial5+trial6+trial7+trial8+trial9+trial10+trial11+(1|center_num), nAGQ=1, family=binomial, data=total)

**Stata package GLLAMM**

gllamm d_unfav pupil2 pupil3 age motor2-motor6 motor9 trial2-trial11, fam(bin) link(logit) i(center_num) adapt

**MIXOR**

binary.OUT

binary.def

1 26 1 19 0.0001 2 0 0 0 0 0 0 5 1 0 0 -1 0 0 0 0

4 2

26

13 14 1 6 7 8 9 10 11 16 17 18 19 20 21 22 23 24 25

0 1

d_unfav

inter

pupil2 pupi3 age motor2 motor3 motor4 motor5 motor6 motor9 trial2

trial3 trial4 trial5 trial6 trial7 trial8 trial9 trial10 trial11

**MLwiN**

GENErate 1 8509 1 c28

WSET

RESP c2

IDEN 2 c4

IDEN 1 c28

RDISt 1 0

LFUN 0

DOFFs 1 C26

ADDT c26

SETV 2 c26

CENT 0

ADDT 'pupil2'

CENT 0

ADDT 'pupil3'

CENT 0

ADDT 'age'

CENT 0

ADDT 'motor2'

CENT 0

ADDT 'motor3'

CENT 0

ADDT 'motor4'

CENT 0

ADDT 'motor5'

CENT 0

ADDT 'motor6'

CENT 0

ADDT 'motor9'

CENT 0

ADDT 'trial2'

CENT 0

ADDT 'trial3'

CENT 0

ADDT 'trial4'

CENT 0

ADDT 'trial5'

CENT 0

ADDT 'trial6'

CENT 0

ADDT 'trial7'

CENT 0

ADDT 'trial8'

CENT 0

ADDT 'trial9'

CENT 0

ADDT 'trial10'

CENT 0

ADDT 'trial11'

set b11 2

set b12 1

set b14 0

**R MCMCglmm**

prior<-list(R = list(V = 1,fix=1 ), G = list(G1 = list(V = 1e-16,nu = -2)))

MCMCglmm(d_unfav ~ pupil2+pupil3+age+motor2+motor3+motor4+motor5

+motor6+motor9+trial2+trial3+trial4+trial5+trial6+trial7+trial8+trial9+trial10+trial11,random=~center_num, family="categorical",

data=total,prior=prior,nitt=10000, thin=1, burnin=3000,verbose = FALSE)

**WinBUGS**

model{

for (i in 1:N) {

agec[i]<-(age[i]-mean(age[]))/sd(age[]) #center age

logit(mu[i])<-beta[1]*pupil2[i]+beta[2]*pupil3[i]+beta[3]*agec[i]+beta[4]*motor2[i]+beta[5]*motor3[i]+beta[6]*motor4[i]+beta[7]*motor5[i]+beta[8]*motor6[i]+beta[9]*motor9[i]+beta[10]*trial2[i]+beta[11]*trial3[i]+beta[12]*trial4[i]+beta[13]*trial5[i]+beta[14]*trial6[i]+beta[15]*trial7[i]+beta[16]*trial8[i]+beta[17]*trial9[i]+beta[18]*trial10[i]+beta[19]*trial11[i]+b[center[i]]

d_unfav[i]~dbin(mu[i],1)

}

for (i in 1:Ncenter){

b[i]~dnorm(beta0,tau)

}

#the following prior distributions were chosen

beta0~dnorm(0,0.0001)

for (j in 1: 19){

beta[j]~dnorm(0,0.0001)

}

sigma~dunif(0.01,100)

tau <- pow(sigma,-1)

}

**Ordinal logistic random effects model**

**SAS procedure nlmixed**

**proc** **nlmixed** data=aa_std tech=newrap qpoints=**5**;

parms beta1=**0**, beta2=-**2**, beta3=-**1**,beta4=**1**,beta5=**1**,beta6=**1**,beta7=**1**, beta8=**1**,beta9=**1**,beta10=**1**,beta11=**1**,beta12=**1**,beta13=**1**,beta14=**1**,beta15=**1**,beta16=**1**,beta17=**1**,beta18=**1**,beta19=**1**,s2b=**1** i1=**1** i2=**1** i3=**1**,i4=**1**;

bounds i2>**0**,i3>**0**,i4>**0**;

eta=beta2*pupil2+beta3*pupil3+beta1*age+beta4*motor2+beta5*motor3+beta6*motor4+beta7*motor5+beta8*motor6+beta9*motor9+beta10*trial2+beta11*trial3+beta12*trial4+beta13*trial5+beta14*trial6+beta15*trial7+beta16*trial8+beta17*trial9+beta18*trial10+beta19*trial11+b0;

if (gos=**1**) then p=**1**/(**1**+exp(-(i1+eta)));

else if (gos=**2**) then p=(**1**/(**1**+exp(-(i1+i2+eta))))-(**1**/(**1**+exp(-(i1+eta))));

else if (gos=**3**) then p=(**1**/(**1**+exp(-(i1+i2+i3+eta))))-(**1**/(**1**+exp(-(i1+i2+eta))));

else if (gos=**4**) then p=(**1**/(**1**+exp(-(i1+i2+i3+i4+eta))))-(**1**/(**1**+exp(-(i1+i2+i3+eta))));

else p=**1**-(**1**/(**1**+exp(-(i1+i2+i3+i4+eta))));

if (p > **1e-8**) then ll = log(p);

else ll = -**1e100**;

model gos ~ general(ll);

random b0 ~ normal(**0**,s2b) subject=center_num out=ord;

estimate 'thresh1' i1;

estimate 'thresh2' i1+i2;

estimate 'thresh3' i1+i2+i3;

estimate 'thresh4' i1+i2+i3+i4;

**run**;

**SAS procedure glimmix**

**proc** **glimmix** data=aa_std method=quad(qpoints=**5**);

class center_num;

model gos=pupil2-pupil3 age motor2-motor6 motor9 trial2-trial11/DIST=MULT LINK=CLOGIT solution;

random intercept/ subject=center_num;

NLOPTIONS MAXIT=**100**;

output out=i1 pred=p resid=r pred(NOBLUP)=p1;

**run**;

**Stata package GLLAMM**

gllamm gos pupil2 pupil3 age motor2-motor6 motor9 trial2-trial11, fam(bin) link(ologit) i(center_num) adapt

**MIXOR**

ordinal.OUT

ordial.def

1 26 1 19 0.0001 5 0 0 0 0 0 0 5 1 0 0 -1 0 0 0 0

4 3

26

13 14 1 6 7 8 9 10 11 16 17 18 19 20 21 22 23 24 25

1 2 3 4 5

gos

inter

pupil2 pupi3 age motor2 motor3 motor4 motor5 motor6 motor9 trial2

trial3 trial4 trial5 trial6 trial7 trial8 trial9 trial10 trial11

**MLwiN**

RCAT "gos"

GENErate 1 8509 1 c28

WSET

RESP c3

IDEN 2 c4

IDEN 1 c28

name c29 'resp' c30 'resp_indicator'

mnom 1 c3 c29 c30 5

set b13 0

iden 1 'resp_indicator'

resp 'resp'

iden 2 c28 3 c4

RDISt 1 5

LFUN 0

DOFFs 1 C26

CENT 0

ADDT 'intercept'

CENT 0

RPAT 1 1 1 1

ADDT 'intercept'

RPAT

SETV 3 c39

FPAR 0 c39

CENT 0

RPAT 1 1 1 1

ADDT 'pupil2'

RPAT

CENT 0

RPAT 1 1 1 1

ADDT 'pupil3'

RPAT

CENT 0

RPAT 1 1 1 1

ADDT 'age'

RPAT

CENT 0

RPAT 1 1 1 1

ADDT 'motor2'

RPAT

CENT 0

RPAT 1 1 1 1

ADDT 'motor3'

RPAT

CENT 0

RPAT 1 1 1 1

ADDT 'motor4'

RPAT

CENT 0

RPAT 1 1 1 1

ADDT 'motor5'

RPAT

CENT 0

RPAT 1 1 1 1

ADDT 'motor6'

RPAT

CENT 0

RPAT 1 1 1 1

ADDT 'motor9'

RPAT

CENT 0

RPAT 1 1 1 1

ADDT 'trial2'

RPAT

CENT 0

RPAT 1 1 1 1

ADDT 'trial3'

RPAT

CENT 0

RPAT 1 1 1 1

ADDT 'trial4'

RPAT

CENT 0

RPAT 1 1 1 1

ADDT 'trial5'

RPAT

CENT 0

RPAT 1 1 1 1

ADDT 'trial6'

RPAT

CENT 0

RPAT 1 1 1 1

ADDT 'trial7'

RPAT

CENT 0

RPAT 1 1 1 1

ADDT 'trial8'

RPAT

CENT 0

RPAT 1 1 1 1

ADDT 'trial9'

RPAT

CENT 0

RPAT 1 1 1 1

ADDT 'trial10'

RPAT

CENT 0

RPAT 1 1 1 1

ADDT 'trial11'

RPAT

set b11 2

set b14 0

set b12 1

**WinBUGS**

model{

for (i in 1:N) {

agec[i]<-(age[i]-mean(age[]))/sd(age[]) #center age

covc[i]<-beta[1]*pupil2[i]+beta[2]*pupil3[i]+beta[3]*agec[i]+beta[4]*motor2[i]+beta[5]*motor3[i]+beta[6]*motor4[i]+beta[7]*motor5[i]+beta[8]*motor6[i]+beta[9]*motor9[i]+beta[10]*trial2[i]+beta[11]*trial3[i]+beta[12]*trial4[i]+beta[13]*trial5[i]+beta[14]*trial6[i]+beta[15]*trial7[i]+beta[16]*trial8[i]+beta[17]*trial9[i]+beta[18]*trial10[i]+beta[19]*trial11[i]+b[center[i]]

for (j in 1:4){logit(f[i,j])<-a[j]+covc[i]}

#cumulative probability of response<=cutpoint

p[i,1]<-f[i,1];p[i,2]<-f[i,2]-f[i,1];p[i,3]<-f[i,3]-f[i,2];p[i,4]<-f[i,4]-f[i,3];p[i,5]<-1-f[i,4];

gos[i]~dcat(p[i,1:5])

}

for (i in 1:Ncenter){

b[i]~dnorm(a[1],tau)

}

a[1] ~ dnorm(0, 1.0E-06)I(,a[2])

a[2] ~ dnorm(0, 1.0E-06)I(a[1],a[3])

a[3] ~ dnorm(0, 1.0E-06)I(a[2],a[4])

a[4] ~ dnorm(0, 1.0E-06)I(a[3],)

for (j in 1: 19){

beta[j]~dnorm(0,0.0001)

}

sigma~dunif(0.01,100)

tau <- pow(sigma,-1)

}

**Cross-classified logistic random effects model**

**R function lmer**

glmer(d_unfav ~ pupil2+ pupil3+ age+ motor2+ motor3+ motor4+ motor5+ motor6+ motor9+(1|total$trial)+(1|center_num), nAGQ=1, family=binomial, data=total)

**SAS procedure glimmix**

**proc** **glimmix** data=aa_std method=quad(qpoints=5);

class center_num trial;

model d_unfav(event=last)=pupil2-pupil3 age motor2-motor6 motor9 / dist=binary solution;

random intercept/ subject=center_num;

random intercept/subject=trial;

output out=i1 pred=p resid=r pred(NOBLUP)=p1;

**run**;

**MLwiN**

RESP c1

IDEN 2 c3

GENErate 1 8509 1 c28

WSET

IDEN 1 c28

RDISt 1 0

LFUN 0

DOFFs 1 C25

ADDT c25

SETV 2 c25

CENT 0

ADDT 'pupil2'

CENT 0

ADDT 'pupil3'

CENT 0

ADDT 'age'

CENT 0

ADDT 'motor2'

CENT 0

ADDT 'motor3'

CENT 0

ADDT 'motor4'

CENT 0

ADDT 'motor5'

CENT 0

ADDT 'motor6'

CENT 0

ADDT 'motor9'

iden 3 'denom'

RCAT "trial"

setx 'denom' 3 'trial' c51-c61 c62

rcon c62

**R MCMCglmm**

MCMCglmm(d_unfav ~ pupil2+pupil3+age+motor2+motor3+motor4+motor5

+motor6+motor9,random=~center_num+trial, family="categorical",

data=total,prior=prior,verbose = FALSE)

**WinBUGS**

model

{

# Level 1 definition

for(i in 1:N) {

d_unfav[i] ~ dbin(p[i],denom[i])

logit(p[i]) <- beta[1] * intercept[i]

+ beta[2] * pupil2[i]

+ beta[3] * pupil3[i]

+ beta[4] * age[i]

+ beta[5] * motor2[i]

+ beta[6] * motor3[i]

+ beta[7] * motor4[i]

+ beta[8] * motor5[i]

+ beta[9] * motor6[i]

+ beta[10] * motor9[i]

+ u2[center_num[i]] * intercept[i]

+ u3[trial[i]] * intercept[i]

}

# Higher level definitions

for (j in 1:n2) {

u2[j] ~ dnorm(0,tau.u2)

}

for (j in 1:n3) {

u3[j] ~ dnorm(0,tau.u3)

}

# Priors for fixed effects

for (k in 1:10) { beta[k] ~ dnorm(0,1.0E-6) }

# Priors for random terms

sigma.u2~dunif(0,20)

sigma2.u2 <- pow(sigma.u2,2)

tau.u2<-1/sigma2.u2

sigma.u3~dunif(0,20)

sigma2.u3 <- pow(sigma.u3,2)

tau.u3<-1/sigma2.u3

}
