# Supplementary material for: Logistic random effects regression models: a comparison of statistical packages for binary and ordinal outcomes
Source: BMC Med Res Methodol. 2011 May 23;11:77. doi: 10.1186/1471-2288-11-77 (PMC3112198; doi:10.1186/1471-2288-11-77)
Supplement: Additional file 3 — IMPACT study: Descriptive statistics of the study population. [file 1471-2288-11-77-S3.DOC]

|  | **TINT** | **TIUS** | **SLIN** | **SAP** | **PEG** | **HITI** | **UK4** | **TCDB** | **SKB** | **EBIC** | **HITII** | **Total** |
| --- | --- | --- | --- | --- | --- | --- | --- | --- | --- | --- | --- | --- |
| **Type** | RCT | RCT | RCT | RCT | RCT | RCT | Obs. | Obs. | RCT | Obs. | RCT |  |
| **Year of study** | 1992-1994 | 1991-1994 | 1994-1996 | 1995-1997 | 1993-1995 | 1987-1989 | 1986-1988 | 1984-1987 | 1996 | 1995 | 1989-1991 |  |
| **No. of patients** | 1131 | 1155 | 409 | 924 | 1574 | 351 | 988 | 667 | 139 | 1005 | 852 | 8509 |
| **No. of centers** | 50 | 36 | 50 | 57 | 29 | 6 | 4 | 4 | 31 | 67 | 21 | 231 |
| **Outcome(GOS)** |  |  |  |  |  |  |  |  |  |  |  |  |
| dead | 278(25%) | 225(22%) | 94(23%) | 212(23%) | 362(24%) | 99(28%) | 359(45%) | 264(44%) | 34(27%) | 281(34%) | 188(23%) | 2396(28%) |
| vegetative | 44(4%) | 42(4%) | 14(3%) | 24(3%) | 114(8%) | 10(3%) | 13(2%) | 34(6%) | 6(5%) | 18(2%) | 32(4%) | 351(4%) |
| severe disability | 134(12%) | 128(12%) | 69(17%) | 142(16%) | 298(20%) | 62(18%) | 146(19%) | 95(16%) | 30(24%) | 123(15%) | 108(13%) | 1335(16%) |
| moderate disability | 171(15%) | 180(17%) | 84(21%) | 174(19%) | 374(25%) | 64(18%) | 130(16%) | 104(17%) | 27(21%) | 159(19%) | 199(24%) | 1666(20%) |
| good recovery | 491(44%) | 466(45%) | 148(36%) | 367(40%) | 362(24%) | 115(33%) | 143(18%) | 107(18%) | 29(23%) | 241(29%) | 292(36%) | 2761(32%) |
| **Predictor(age)** |  |  |  |  |  |  |  |  |  |  |  |  |
| Median(IQ range) | 30(21-45) | 30(23-41) | 28(21-43) | 32(23-47) | 27(20-38) | 34(21-47) | 36(22-55) | 26(21-40) | 27(20-39) | 37.5(24-59) | 33(22-49) | 30(21-45) |
| **Predictor(motor)** |  |  |  |  |  |  |  |  |  |  |  |  |
| none | 5(0%) | 9(1%) | 0(0%) | 141(15%) | 475(32%) | 122(35%) | 113(14%) | 136(23%) | 34(27%) | 150(18%) | 210(26%) | 1395(16%) |
| extension | 136(12%) | 143(14%) | 55(13%) | 123(13%) | 180(12%) | 41(12%) | 85(11%) | 107(18%) | 22(18%) | 80(10%) | 70(9%) | 1042(12%) |
| abnormal flexion | 237(21%) | 132(13%) | 91(22%) | 143(16%) | 165(11%) | 45(13%) | 37(5%) | 74(12%) | 14(11%) | 55(7%) | 92(11%) | 1085(13%) |
| normal flexion | 327(29%) | 300(29%) | 127(31%) | 223(24%) | 334(22%) | 56(16%) | 141(18%) | 122(20%) | 16(13%) | 113(14%) | 181(22%) | 1940(23%) |
| localises | 384(34%) | 406(39%) | 134(33%) | 286(31%) | 309(21%) | 77(22%) | 191(24%) | 113(19%) | 21(17%) | 182(22%) | 199(24%) | 2302(27%) |
| obeys command | 29(3%) | 51(5%) | 2(1%) | 0(0%) | 47(3%) | 0(0%) | 30(4%) | 21(4%) | 2(2%) | 99(12%) | 8(1%) | 289(3%) |
| untestable & not available | 0(0%) | 0(0%) | 0(0%) | 3(0%) | 0(0%) | 9(3%) | 194(25%) | 31(6%) | 17(14%) | 143(18%) | 59(7%) | 456(5%) |
| **Predictor(pupil)** |  |  |  |  |  |  |  |  |  |  |  |  |
| both side positive | 806(72%) | 703(68%) | 315(77%) | 619(67%) | 784(52%) | 232(66%) | 427(54%) | 300(50%) | 70(56%) | 535(65%) | 585(71%) | 5376(63%) |
| one side positive | 177(16%) | 118(11%) | 79(19%) | 178(19%) | 156(10%) | 53(15%) | 115(15%) | 55(9%) | 35(28%) | 79(10%) | 99(12%) | 1144(13%) |
| both side negative | 135(12%) | 220(21%) | 15(4%) | 122(13%) | 570(38%) | 65(19%) | 249(32%) | 249(41%) | 21(17%) | 208(25%) | 135(17%) | 1989(23%) |
