# Supplementary material for: Logistic random effects regression models: a comparison of statistical packages for binary and ordinal outcomes
Source: BMC Med Res Methodol. 2011 May 23;11:77. doi: 10.1186/1471-2288-11-77 (PMC3112198; doi:10.1186/1471-2288-11-77)
Supplement: Additional file 4 — IMPACT study: Results of the binary model in case 1 (full data set). * The variance of the random effects with its standard error is given. [file 1471-2288-11-77-S4.DOC]

|  | R(lme4) | | | GLLAMM | | GLIMMIX | | NLMIXED | | MLwiN([R]IGLS) | | MIXOR | | WinBUGS | | MLwiN(MCMC) | | MCMCglmm | | MCMC | |
| --- | --- | --- | --- | --- | --- | --- | --- | --- | --- | --- | --- | --- | --- | --- | --- | --- | --- | --- | --- | --- | --- |
| Computing time | 34s | | | 7min | | 9s | | 15min | | 2s | | 30s | | 14min | | 4min | | 2min | | 37h | |
| Random Effects | Variance:  0.101 | | | Variance:  0.102(0.027) | | Variance:  0.107(0.027) | | Variance:  0.102(0.027) | | Variance:  0.101(0.025) | | Variance:  0.102(0.032) | | Variance:  0.119(0.030) | | Variance:  0.113(0.030) | | Variance:  0.110(0.031) | | Variance:  0.160(0.034) | |
| Fixed Effects | covar | **Coef** | SE | **Coef** | SE | **Coef** | SE | **Coef** | SE | **Coef** | SE | **Coef** | SE | **Coef** | SE | **Coef** | SE | **Coef** | SE | **Coef** | SE |
|  | const | **-0.014** | 0.114 | **-0.014** | 0.114 | **-0.014** | 0.114 | **-0.014** | 0.114 | **-0.014** | 0.114 | **-0.014** | 0.126 | **-0.026** | 0.115 | **-0.003** | 0.110 | **-0.019** | 0.121 | **-0.103** | 0.099 |
|  | pupil2 | **0.656** | 0.074 | **0.656** | 0.074 | **0.65** | 0.074 | **0.656** | 0.075 | **0.657** | 0.074 | **0.656** | 0.089 | **0.659** | 0.075 | **0.656** | 0.075 | **0.674** | 0.072 | **0.666** | 0.071 |
|  | pupil3 | **1.404** | 0.069 | **1.404** | 0.07 | **1.392** | 0.069 | **1.404** | 0.07 | **1.405** | 0.069 | **1.404** | 0.075 | **1.410** | 0.069 | **1.406** | 0.068 | **1.434** | 0.068 | **1.424** | 0.069 |
|  | age | **0.623** | 0.028 | **0.623** | 0.028 | **0.618** | 0.028 | **0.623** | 0.028 | **0.623** | 0.028 | **0.623** | 0.029 | **0.626** | 0.028 | **0.625** | 0.029 | **0.636** | 0.028 | **0.630** | 0.029 |
|  | motor2 | **0.618** | 0.106 | **0.618** | 0.106 | **0.612** | 0.105 | **0.618** | 0.106 | **0.618** | 0.106 | **0.618** | 0.126 | **0.623** | 0.106 | **0.617** | 0.104 | **0.623** | 0.110 | **0.654** | 0.103 |
|  | motor3 | **-0.154** | 0.097 | **-0.154** | 0.097 | **-0.153** | 0.097 | **-0.154** | 0.097 | **-0.154** | 0.097 | **-0.154** | 0.101 | **-0.152** | 0.098 | **-0.158** | 0.096 | **-0.159** | 0.105 | **-0.131** | 0.096 |
|  | motor4 | **-0.782** | 0.086 | **-0.782** | 0.086 | **-0.775** | 0.086 | **-0.782** | 0.087 | **-0.782** | 0.086 | **-0.782** | 0.103 | **-0.781** | 0.088 | **-0.786** | 0.084 | **-0.811** | 0.089 | **-0.757** | 0.076 |
|  | motor5 | **-1.404** | 0.088 | **-1.404** | 0.089 | **-1.394** | 0.088 | **-1.404** | 0.089 | **-1.405** | 0.088 | **-1.404** | 0.108 | **-1.409** | 0.090 | **-1.412** | 0.086 | **-1.449** | 0.097 | **-1.394** | 0.070 |
|  | motor6 | **-1.591** | 0.166 | **-1.591** | 0.167 | **-1.577** | 0.166 | **-1.591** | 0.167 | **-1.592** | 0.166 | **-1.591** | 0.186 | **-1.598** | 0.168 | **-1.602** | 0.168 | **-1.642** | 0.177 | **-1.593** | 0.166 |
|  | motor9 | **-0.534** | 0.136 | **-0.534** | 0.136 | **-0.529** | 0.136 | **-0.534** | 0.136 | **-0.534** | 0.136 | **-0.534** | 0.156 | **-0.535** | 0.136 | **-0.536** | 0.136 | **-0.561** | 0.150 | **-0.533** | 0.129 |
|  | trial2 | **-0.073** | 0.125 | **-0.073** | 0.126 | **-0.071** | 0.126 | **-0.073** | 0.126 | **-0.073** | 0.125 | **-0.073** | 0.132 | **-0.061** | 0.129 | **-0.081** | 0.121 | **-0.058** | 0.131 | **-0.007** | 0.115 |
|  | trial3 | **0.218** | 0.139 | **0.217** | 0.139 | **0.216** | 0.139 | **0.218** | 0.139 | **0.218** | 0.138 | **0.217** | 0.136 | **0.222** | 0.140 | **0.210** | 0.136 | **0.229** | 0.141 | **0.240** | 0.139 |
|  | trial4 | **-0.192** | 0.116 | **-0.192** | 0.117 | **-0.189** | 0.117 | **-0.192** | 0.117 | **-0.192** | 0.116 | **-0.192** | 0.099 | **-0.184** | 0.117 | **-0.195** | 0.115 | **-0.174** | 0.122 | **-0.116** | 0.128 |
|  | trial5 | **0.107** | 0.114 | **0.107** | 0.115 | **0.107** | 0.115 | **0.107** | 0.115 | **0.107** | 0.114 | **0.107** | 0.128 | **0.119** | 0.117 | **0.099** | 0.114 | **0.114** | 0.117 | **0.184** | 0.112 |
|  | trial6 | **-0.039** | 0.173 | **-0.039** | 0.174 | **-0.039** | 0.174 | **-0.039** | 0.174 | **-0.039** | 0.173 | **-0.039** | 0.202 | **-0.034** | 0.175 | **-0.046** | 0.172 | **-0.048** | 0.187 | **0.049** | 0.188 |
|  | trial7 | **0.686** | 0.170 | **0.686** | 0.17 | **0.68** | 0.171 | **0.686** | 0.17 | **0.687** | 0.17 | **0.686** | 0.151 | **0.693** | 0.172 | **0.680** | 0.172 | **0.704** | 0.184 | **0.755** | 0.182 |
|  | trial8 | **0.672** | 0.176 | **0.672** | 0.176 | **0.665** | 0.177 | **0.672** | 0.176 | **0.673** | 0.176 | **0.672** | 0.175 | **0.682** | 0.181 | **0.652** | 0.172 | **0.691** | 0.182 | **0.744** | 0.198 |
|  | trial9 | **0.373** | 0.231 | **0.373** | 0.232 | **0.368** | 0.231 | **0.373** | 0.232 | **0.373** | 0.231 | **0.373** | 0.229 | **0.382** | 0.234 | **0.368** | 0.232 | **0.382** | 0.248 | **0.408** | 0.223 |
|  | trial10 | **0.090** | 0.123 | **0.09** | 0.123 | **0.09** | 0.123 | **0.09** | 0.123 | **0.09** | 0.123 | **0.090** | 0.112 | **0.099** | 0.124 | **0.083** | 0.118 | **0.097** | 0.127 | **0.149** | 0.125 |
|  | trial11 | **-0.239** | 0.125 | **-0.238** | 0.127 | **-0.233** | 0.126 | **-0.238** | 0.127 | **-0.239** | 0.125 | **-0.238** | 0.144 | **-0.225** | 0.127 | **-0.239** | 0.123 | **-0.230** | 0.134 | **-0.128** | 0.121 |
