# Supplementary material for: Logistic random effects regression models: a comparison of statistical packages for binary and ordinal outcomes
Source: BMC Med Res Methodol. 2011 May 23;11:77. doi: 10.1186/1471-2288-11-77 (PMC3112198; doi:10.1186/1471-2288-11-77)
Supplement: Additional file 5 — IMPACT study: Results from the ordinal model in case 1 (full data set). * The variance of the random effects with its standard error is given [file 1471-2288-11-77-S5.DOC]

|  | GLLAMM | | | GLIMMIX | | NLMIXED | | MLwiN([R]IGLS) | | MIXOR | | WinBUGS | | MLwiN(MCMC) | |
| --- | --- | --- | --- | --- | --- | --- | --- | --- | --- | --- | --- | --- | --- | --- | --- |
| Computing time | 11min | | | 11s | | 24min | | 6s | | 3min | | 8h | | 15min | |
| Random  Effects | Variance:  0.085(0.020) | | | Variance:  0.090(0.021) | | Variance:  0.085(0.020) | | Variance:  0.085(0.019) | | Variance:  0.085(0.024) | | Variance:  0.096(0.022) | | Variance:  0.093(0.022) | |
| Fixed  Effects | covar | **Coef** | SE | **Coef** | SE | **Coef** | SE | **Coef** | SE | **Coef** | SE | **Coef** | SE | **Coef** | SE |
|  | pupil2 | **0.705** | 0.062 | **0.702** | 0.062 | **0.705** | 0.062 | **0.707** | 0.062 | **0.705** | 0.082 | **0.703** | 0.062 | **0.708** | 0.063 |
|  | pupil3 | **1.401** | 0.057 | **1.396** | 0.057 | **1.401** | 0.057 | **1.406** | 0.057 | **1.401** | 0.062 | **1.405** | 0.057 | **1.406** | 0.057 |
|  | age | **0.591** | 0.023 | **0.588** | 0.023 | **0.591** | 0.023 | **0.592** | 0.023 | **0.591** | 0.027 | **0.551** | 0.023 | **0.592** | 0.023 |
|  | motor2 | **0.277** | 0.083 | **0.275** | 0.083 | **0.277** | 0.083 | **0.279** | 0.083 | **0.277** | 0.091 | **0.276** | 0.082 | **0.282** | 0.086 |
|  | motor3 | **-0.296** | 0.081 | **-0.295** | 0.081 | **-0.296** | 0.081 | **-0.296** | 0.081 | **-0.296** | 0.077 | **-0.305** | 0.080 | **-0.292** | 0.084 |
|  | motor4 | **-0.846** | 0.072 | **-0.843** | 0.072 | **-0.846** | 0.072 | **-0.848** | 0.072 | **-0.846** | 0.074 | **-0.847** | 0.072 | **-0.843** | 0.074 |
|  | motor5 | **-1.369** | 0.073 | **-1.365** | 0.073 | **-1.369** | 0.073 | **-1.373** | 0.073 | **-1.369** | 0.080 | **-1.368** | 0.073 | **-1.367** | 0.076 |
|  | motor6 | **-1.572** | 0.137 | **-1.565** | 0.133 | **-1.572** | 0.137 | **-1.577** | 0.133 | **-1.572** | 0.156 | **-1.567** | 0.137 | **-1.574** | 0.138 |
|  | motor9 | **-0.630** | 0.111 | **-0.628** | 0.112 | **-0.630** | 0.111 | **-0.632** | 0.112 | **-0.630** | 0.115 | **-0.640** | 0.112 | **-0.629** | 0.112 |
|  | trial2 | **-0.067** | 0.107 | **-0.066** | 0.106 | **-0.067** | 0.107 | **-0.067** | 0.105 | **-0.067** | 0.112 | **-0.075** | 0.109 | **-0.054** | 0.113 |
|  | trial3 | **0.252** | 0.117 | **0.251** | 0.116 | **0.252** | 0.117 | **0.253** | 0.116 | **0.252** | 0.114 | **0.245** | 0.117 | **0.260** | 0.120 |
|  | trial4 | **-0.122** | 0.099 | **-0.120** | 0.098 | **-0.122** | 0.099 | **-0.122** | 0.097 | **-0.122** | 0.083 | **-0.121** | 0.099 | **-0.111** | 0.103 |
|  | trial5 | **0.189** | 0.097 | **0.190** | 0.097 | **0.189** | 0.097 | **0.190** | 0.096 | **0.189** | 0.106 | **0.177** | 0.098 | **0.204** | 0.103 |
|  | trial6 | **0.051** | 0.146 | **0.051** | 0.147 | **0.051** | 0.146 | **0.051** | 0.146 | **0.051** | 0.138 | **0.083** | 0.147 | **0.062** | 0.149 |
|  | trial7 | **0.772** | 0.142 | **0.768** | 0.144 | **0.772** | 0.142 | **0.775** | 0.143 | **0.772** | 0.132 | **0.783** | 0.144 | **0.781** | 0.145 |
|  | trial8 | **0.901** | 0.148 | **0.900** | 0.149 | **0.901** | 0.148 | **0.904** | 0.148 | **0.900** | 0.259 | **0.888** | 0.150 | **0.917** | 0.151 |
|  | trial9 | **0.341** | 0.190 | **0.339** | 0.193 | **0.341** | 0.190 | **0.342** | 0.193 | **0.341** | 0.183 | **0.343** | 0.192 | **0.352** | 0.195 |
|  | trial10 | **0.265** | 0.102 | **0.264** | 0.102 | **0.265** | 0.102 | **0.266** | 0.101 | **0.265** | 0.088 | **0.302** | 0.102 | **0.275** | 0.105 |
|  | trial11 | **-0.047** | 0.106 | **-0.044** | 0.105 | **-0.047** | 0.106 | **-0.047** | 0.104 | **-0.047** | 0.092 | **-0.030** | 0.106 | **-0.033** | 0.111 |
|  | Inter1 | **-1.190** | 0.098 | **-1.188** | 0.098 | **-1.190** | 0.098 | **-1.197** | 0.097 | **-1.190** | 0.094 | **-1.186** | 0.098 | **-1.208** | 0.111 |
|  | Inter2 | **-0.931** | 0.098 | **-0.930** | 0.097 | **-0.931** | 0.098 | **-0.937** | 0.097 | **-0.931** | 0.106 | **-0.928** | 0.098 | **-0.949** | 0.111 |
|  | Inter3 | **-0.040** | 0.098 | **-0.040** | 0.097 | **-0.040** | 0.098 | **-0.041** | 0.096 | **-0.040** | 0.117 | **-0.042** | 0.100 | **-0.056** | 0.109 |
|  | Inter4 | **1.026** | 0.098 | **1.025** | 0.097 | **1.026** | 0.098 | **1.031** | 0.097 | **1.026** | 0.121 | **1.007** | 0.103 | **1.012** | 0.109 |
