# Supplementary material for: Logistic random effects regression models: a comparison of statistical packages for binary and ordinal outcomes
Source: BMC Med Res Methodol. 2011 May 23;11:77. doi: 10.1186/1471-2288-11-77 (PMC3112198; doi:10.1186/1471-2288-11-77)
Supplement: Additional file 6 — IMPACT study: Results from the binary model in case 2 (sample 1). * The variance of the random effects with its standard error is given [file 1471-2288-11-77-S6.DOC]

|  | R(lme4) | | | GLLAMM | | GLIMMIX | | NLMIXED | | MLwiN([R]IGLS) | | MIXOR | | WinBUGS | | MLwiN(MCMC) | | MCMCglmm | |
| --- | --- | --- | --- | --- | --- | --- | --- | --- | --- | --- | --- | --- | --- | --- | --- | --- | --- | --- | --- |
| Computing time | 1s | | | 1m | | 1s | | 49s | | 1s | | 1s | | 3min | | 1min | | 9s | |
| Random Effects | Variance:  0.046 | | | Variance:  0.051(0.150) | | Variance:  0.051(0.150) | | Variance:  0.051(0.150) | | Variance:  0.047(0.127) | | Variance:  0.051(0.237) | | Variance:  0.450(0.336) | | Variance:  0.348(0.289) | | Variance:  0.279(0.275) | |
| Fixed Effects | covar | **Coef** | SE | **Coef** | SE | **Coef** | SE | **Coef** | SE | **Coef** | SE | **Coef** | SE | **Coef** | SE | **Coef** | SE | **Coef** | SE |
|  | const | **-0.157** | 0.455 | **-0.156** | 0.458 | **-0.157** | 0.458 | **-0.156** | 0.458 | **-0.157** | 0.455 | **-0.156** | 0.598 | **-0.093** | 0.621 | **-0.106** | 0.420 | **-0.175** | 0.525 |
|  | pupil2 | **0.432** | 0.320 | **0.431** | 0.321 | **0.431** | 0.321 | **0.431** | 0.321 | **0.432** | 0.320 | **0.431** | 0.343 | **0.438** | 0.352 | **0.447** | 0.340 | **0.456** | 0.356 |
|  | pupil3 | **1.357** | 0.284 | **1.359** | 0.289 | **1.359** | 0.289 | **1.359** | 0.289 | **1.358** | 0.284 | **1.359** | 0.354 | **1.512** | 0.314 | **1.504** | 0.314 | **1.553** | 0.303 |
|  | age | **0.638** | 0.115 | **0.638** | 0.117 | **0.638** | 0.117 | **0.638** | 0.117 | **0.638** | 0.115 | **0.638** | 0.146 | **0.706** | 0.128 | **0.699** | 0.125 | **0.727** | 0.126 |
|  | motor2 | **0.040** | 0.435 | **0.039** | 0.437 | **0.039** | 0.437 | **0.039** | 0.437 | **0.040** | 0.435 | **0.039** | 0.591 | **0.032** | 0.496 | **0.054** | 0.449 | **0.112** | 0.484 |
|  | motor3 | **-0.066** | 0.432 | **-0.068** | 0.438 | **-0.068** | 0.438 | **-0.068** | 0.438 | **-0.066** | 0.432 | **-0.068** | 0.574 | **-0.169** | 0.505 | **-0.124** | 0.441 | **-0.136** | 0.496 |
|  | motor4 | **-0.772** | 0.369 | **-0.773** | 0.375 | **-0.773** | 0.375 | **-0.774** | 0.375 | **-0.772** | 0.369 | **-0.774** | 0.506 | **-0.914** | 0.452 | **-0.860** | 0.373 | **-0.863** | 0.417 |
|  | motor5 | **-0.928** | 0.361 | **-0.930** | 0.368 | **-0.930** | 0.368 | **-0.930** | 0.368 | **-0.928** | 0.361 | **-0.930** | 0.499 | **-1.076** | 0.445 | **-1.032** | 0.373 | **-1.089** | 0.407 |
|  | motor6 | **-1.600** | 0.733 | **-1.602** | 0.737 | **-1.601** | 0.737 | **-1.602** | 0.737 | **-1.600** | 0.733 | **-1.602** | 0.967 | **-1.825** | 0.822 | **-1.794** | 0.781 | **-1.868** | 0.809 |
|  | motor9 | **0.469** | 0.580 | **0.469** | 0.582 | **0.469** | 0.582 | **0.469** | 0.582 | **0.469** | 0.580 | **0.469** | 0.749 | **0.526** | 0.650 | **0.547** | 0.628 | **0.529** | 0.599 |
|  | trial2 | **-0.315** | 0.446 | **-0.315** | 0.448 | **-0.315** | 0.448 | **-0.315** | 0.448 | **-0.315** | 0.446 | **-0.315** | 0.457 | **-0.365** | 0.527 | **-0.380** | 0.490 | **-0.283** | 0.518 |
|  | trial3 | **0.634** | 0.510 | **0.635** | 0.513 | **0.636** | 0.513 | **0.635** | 0.513 | **0.634** | 0.510 | **0.635** | 0.561 | **0.712** | 0.588 | **0.667** | 0.543 | **0.729** | 0.577 |
|  | trial4 | **-0.226** | 0.423 | **-0.226** | 0.425 | **-0.226** | 0.425 | **-0.226** | 0.425 | **-0.226** | 0.423 | **-0.226** | 0.489 | **-0.258** | 0.511 | **-0.285** | 0.442 | **-0.229** | 0.445 |
|  | trial5 | **0.542** | 0.405 | **0.542** | 0.407 | **0.542** | 0.408 | **0.542** | 0.408 | **0.542** | 0.405 | **0.542** | 0.428 | **0.538** | 0.496 | **0.515** | 0.423 | **0.590** | 0.460 |
|  | trial6 | **0.077** | 0.648 | **0.078** | 0.651 | **0.078** | 0.651 | **0.078** | 0.651 | **0.078** | 0.648 | **0.077** | 0.776 | **0.079** | 0.769 | **0.043** | 0.691 | **0.177** | 0.739 |
|  | trial7 | **0.680** | 0.492 | **0.681** | 0.497 | **0.682** | 0.497 | **0.682** | 0.497 | **0.680** | 0.492 | **0.681** | 0.638 | **0.718** | 0.611 | **0.687** | 0.577 | **0.843** | 0.578 |
|  | trial8 | **0.628** | 0.504 | **0.627** | 0.507 | **0.627** | 0.507 | **0.627** | 0.507 | **0.628** | 0.504 | **0.627** | 0.499 | **0.582** | 0.639 | **0.528** | 0.583 | **0.619** | 0.655 |
|  | trial9 | **1.553** | 0.927 | **1.555** | 0.932 | **1.555** | 0.932 | **1.555** | 0.932 | **1.554** | 0.927 | **1.555** | 1.402 | **1.800** | 1.097 | **1.787** | 1.047 | **1.963** | 1.052 |
|  | trial10 | **-0.017** | 0.458 | **-0.018** | 0.462 | **-0.018** | 0.462 | **-0.018** | 0.462 | **-0.017** | 0.458 | **-0.018** | 0.556 | **-0.126** | 0.535 | **-0.133** | 0.468 | **-0.110** | 0.543 |
|  | trial11 | **0.093** | 0.442 | **0.094** | 0.444 | **0.094** | 0.444 | **0.094** | 0.444 | **0.093** | 0.442 | **0.093** | 0.580 | **0.124** | 0.541 | **0.099** | 0.468 | **0.154** | 0.509 |
