# Supplementary material for: Logistic random effects regression models: a comparison of statistical packages for binary and ordinal outcomes
Source: BMC Med Res Methodol. 2011 May 23;11:77. doi: 10.1186/1471-2288-11-77 (PMC3112198; doi:10.1186/1471-2288-11-77)
Supplement: Additional file 7 — IMPACT study: Results from the binary model in case 3 (sample 2). * The variance of the random effects with its standard error is given [file 1471-2288-11-77-S7.DOC]

|  | R(lme4) | | | GLLAMM | | GLIMMIX | | NLMIXED | | MLwiN([R]IGLS) | | MIXOR | | WinBUGS | | MLwiN(MCMC) | |
| --- | --- | --- | --- | --- | --- | --- | --- | --- | --- | --- | --- | --- | --- | --- | --- | --- | --- |
| Computing time | 1s | | | 40s | | 1s | | 14s | | 1s | | 1s | | 75min | | 30min | |
| Random Effects | Variance:  0 | | | Variance:  0(0) | | Variance:  0( ) | | Variance:  0( ) | | Variance:  0(0) | | Variance: | | Variance:  28.040(22.130) | | Variance:  19.892(17.236) | |
| Fixed Effects | covar | **Coef** | SE | **Coef** | SE | **Coef** | SE | **Coef** | SE | **Coef** | SE | **Coef** | SE | **Coef** | SE | **Coef** | SE |
|  | const | **-0.549** | 0.618 | **-0.549** | 0.618 | **-0.549** | 0.618 | **-0.549** | 0.618 | **-0.549** | 0.618 | **-0.549** | 0.674 | **-1.725** | 1.644 | **-1.448** | 1.473 |
|  | pupil2 | **0.322** | 0.451 | **0.322** | 0.451 | **0.322** | 0.451 | **0.322** | 0.451 | **0.322** | 0.451 | **0.322** | 0.472 | **1.946** | 1.184 | **1.647** | 1.109 |
|  | pupil3 | **1.187** | 0.408 | **1.187** | 0.408 | **1.187** | 0.408 | **1.187** | 0.408 | **1.187** | 0.408 | **1.187** | 0.471 | **3.141** | 1.148 | **2.774** | 1.085 |
|  | age | **0.729** | 0.175 | **0.729** | 0.174 | **0.729** | 0.175 | **0.729** | 0.175 | **0.729** | 0.174 | **0.729** | 0.189 | **2.195** | 0.719 | **1.913** | 0.675 |
|  | motor2 | **1.231** | 0.653 | **1.231** | 0.652 | **1.231** | 0.653 | **1.231** | 0.653 | **1.231** | 0.652 | **1.231** | 0.791 | **3.933** | 1.851 | **3.400** | 1.713 |
|  | motor3 | **0.226** | 0.562 | **0.226** | 0.562 | **0.226** | 0.562 | **0.226** | 0.562 | **0.226** | 0.562 | **0.226** | 0.608 | **1.571** | 1.332 | **1.304** | 1.231 |
|  | motor4 | **-0.714** | 0.539 | **-0.714** | 0.539 | **-0.714** | 0.539 | **-0.714** | 0.539 | **-0.714** | 0.539 | **-0.714** | 0.569 | **-1.553** | 1.128 | **-1.434** | 1.062 |
|  | motor5 | **-1.700** | 0.533 | **-1.700** | 0.533 | **-1.700** | 0.533 | **-1.700** | 0.533 | **-1.700** | 0.533 | **-1.700** | 0.624 | **-3.742** | 1.312 | **-3.382** | 1.243 |
|  | motor6 | **-1.238** | 0.860 | **-1.238** | 0.859 | **-1.238** | 0.860 | **-1.238** | 0.860 | **-1.238** | 0.859 | **-1.238** | 0.898 | **-0.484** | 1.978 | **-0.800** | 1.790 |
|  | motor9 | **-1.423** | 0.775 | **-1.423** | 0.775 | **-1.423** | 0.775 | **-1.423** | 0.775 | **-1.423** | 0.775 | **-1.423** | 0.917 | **-0.969** | 1.415 | **-1.107** | 1.333 |
|  | trial2 | **0.433** | 0.579 | **0.433** | 0.579 | **0.433** | 0.579 | **0.433** | 0.579 | **0.433** | 0.579 | **0.433** | 0.606 | **-0.900** | 1.883 | **-0.600** | 1.650 |
|  | trial3 | **1.083** | 0.781 | **1.083** | 0.781 | **1.083** | 0.781 | **1.083** | 0.781 | **1.083** | 0.781 | **1.083** | 1.021 | **1.381** | 2.091 | **1.292** | 1.847 |
|  | trial4 | **-0.523** | 0.692 | **-0.523** | 0.692 | **-0.523** | 0.692 | **-0.523** | 0.692 | **-0.523** | 0.692 | **-0.523** | 0.671 | **-2.070** | 1.898 | **-1.813** | 1.715 |
|  | trial5 | **0.580** | 0.588 | **0.580** | 0.588 | **0.580** | 0.588 | **0.580** | 0.588 | **0.580** | 0.588 | **0.580** | 0.606 | **1.936** | 1.845 | **1.598** | 1.601 |
|  | trial6 | **1.100** | 0.853 | **1.100** | 0.853 | **1.100** | 0.853 | **1.100** | 0.853 | **1.100** | 0.853 | **1.100** | 1.524 | **2.633** | 2.053 | **2.398** | 1.876 |
|  | trial7 | **1.769** | 0.678 | **1.769** | 0.678 | **1.769** | 0.678 | **1.769** | 0.678 | **1.769** | 0.678 | **1.769** | 1.776 | **1.657** | 1.919 | **1.678** | 1.744 |
|  | trial8 | **0.714** | 0.671 | **0.714** | 0.671 | **0.714** | 0.671 | **0.714** | 0.671 | **0.714** | 0.671 | **0.714** | 1.356 | **-0.515** | 2.412 | **-0.226** | 2.135 |
|  | trial9 | **1.805** | 1.033 | **1.805** | 1.033 | **1.805** | 1.033 | **1.805** | 1.033 | **1.805** | 1.033 | **1.805** | 0.923 | **4.520** | 3.447 | **3.968** | 3.005 |
|  | trial10 | **0.689** | 0.655 | **0.689** | 0.655 | **0.689** | 0.655 | **0.689** | 0.655 | **0.689** | 0.655 | **0.689** | 0.745 | **0.727** | 1.579 | **0.748** | 1.425 |
|  | trial11 | **0.322** | 0.668 | **0.322** | 0.668 | **0.322** | 0.668 | **0.322** | 0.668 | **0.322** | 0.668 | **0.322** | 0.741 | **0.412** | 1.405 | **0.382** | 1.296 |
