# Supplementary material for: Logistic random effects regression models: a comparison of statistical packages for binary and ordinal outcomes
Source: BMC Med Res Methodol. 2011 May 23;11:77. doi: 10.1186/1471-2288-11-77 (PMC3112198; doi:10.1186/1471-2288-11-77)
Supplement: Additional file 8 — IMPACT study: Results from the cross-classified model in case 4 (full data set). * The variance of the random effects with its standard error is given [file 1471-2288-11-77-S8.DOC]

|  | | R(lme4) | | | GLIMMIX | | MLwiN([R]IGLS) | | WinBUGS | | MLwiN(MCMC) | | MCMCglmm | |
| --- | --- | --- | --- | --- | --- | --- | --- | --- | --- | --- | --- | --- | --- | --- |
| Computing time | | 18s | | | 3s | | 5s | | 17min | | 3min | | 3min | |
| Random Effects | Center | 0.116 | | | 0.113(0.028) | | 0.116(0.028) | | 0.119(0.031) | | 0.120(0.032) | | 0.106(0.031) | |
|  | Trial | 0.067 | | | 0.075(0.042) | | 0.067(0.035) | | 0.114(0.079) | | 0.095(0.065) | | 0.094(0.113) | |
| Fixed Effects | | covar | **Coef** | SE | **Coef** | SE | **Coef** | SE | **Coef** | SE | **Coef** | SE | **Coef** | SE |
|  |  | const | **0.105** | 0.111 | **0.107** | 0.114 | **0.105** | 0.112 | **0.126** | 0.132 | **0.14** | 0.127 | **0.110** | 0.140 |
|  |  | pupil2 | **0.657** | 0.074 | **0.650** | 0.074 | **0.658** | 0.074 | **0.656** | 0.075 | **0.657** | 0.075 | **0.681** | 0.080 |
|  |  | pupil3 | **1.411** | 0.069 | **1.396** | 0.069 | **1.412** | 0.069 | **1.412** | 0.069 | **1.413** | 0.07 | **1.436** | 0.073 |
|  |  | age | **0.623** | 0.028 | **0.617** | 0.028 | **0.623** | 0.028 | **0.624** | 0.028 | **0.624** | 0.027 | **0.635** | 0.028 |
|  |  | motor2 | **0.620** | 0.105 | **0.613** | 0.105 | **0.620** | 0.105 | **0.623** | 0.108 | **0.61** | 0.106 | **0.631** | 0.106 |
|  |  | motor3 | **-0.155** | 0.097 | **-0.154** | 0.097 | **-0.156** | 0.097 | **-0.152** | 0.100 | **-0.165** | 0.1 | **-0.157** | 0.096 |
|  |  | motor4 | **-0.782** | 0.086 | **-0.773** | 0.086 | **-0.782** | 0.086 | **-0.780** | 0.087 | **-0.793** | 0.093 | **-0.798** | 0.089 |
|  |  | motor5 | **-1.406** | 0.088 | **-1.392** | 0.087 | **-1.408** | 0.088 | **-1.406** | 0.091 | **-1.417** | 0.093 | **-1.432** | 0.087 |
|  |  | motor6 | **-1.579** | 0.166 | **-1.563** | 0.165 | **-1.581** | 0.166 | **-1.584** | 0.169 | **-1.602** | 0.167 | **-1.617** | 0.168 |
|  |  | motor9 | **-0.502** | 0.135 | **-0.498** | 0.135 | **-0.502** | 0.135 | **-0.509** | 0.136 | **-0.52** | 0.141 | **-0.524** | 0.132 |
