# Supplementary material for: Logistic random effects regression models: a comparison of statistical packages for binary and ordinal outcomes
Source: BMC Med Res Methodol. 2011 May 23;11:77. doi: 10.1186/1471-2288-11-77 (PMC3112198; doi:10.1186/1471-2288-11-77)
Supplement: Additional file 9 — Impact of variance component priors on the posterior means in WinBUGS and MLwiN in case 3 (sample 2). * The variance of the random effects with its standard error is given. * The first three uniform distributions in WinBUGS are for the standard deviation of the random effects and the rest four prior distributions are for the variance of the random effect [file 1471-2288-11-77-S9.DOC]

|  | WinBUGS | | | | | | | | | | | MLwiN (MCMC) | | | |
| --- | --- | --- | --- | --- | --- | --- | --- | --- | --- | --- | --- | --- | --- | --- | --- |
| Distribution | Uniform(0,1) | | | Uniform(0,10) | | Uniform(0,100) | | Uniform(0,10^6^) | | IG (0.001,0.001) | | IG (0.001,0.001) | | Uniform(0,infinity) | |
| Random Effects | Variance:  0.489(0.312) | | | Variance:  26.450(18.250) | | Variance:  28.040(22.130) | | Variance:  36.950(29.120) | | Variance:  20.310(17.570)) | | Variance:  19.892(17.236) | | Variance:  36.954(28.406) | |
| Fixed Effects | covar | **Coef** | SE | **Coef** | SE | **Coef** | SE | **Coef** | SE | **Coef** | SE | **Coef** | SE | **Coef** | SE |
|  | const | **-0.599** | 0.695 | **-1.720** | 1.610 | **-1.725** | 1.644 | **-1.942** | 1.830 | **-1.463** | 1.482 | **-1.448** | 1.473 | **-1.970** | 1.833 |
|  | pupil2 | **0.435** | 0.510 | **1.927** | 1.161 | **1.946** | 1.184 | **2.177** | 1.267 | **1.662** | 1.121 | **1.647** | 1.109 | **2.198** | 1.270 |
|  | pupil3 | **1.384** | 0.461 | **3.098** | 1.101 | **3.141** | 1.148 | **3.455** | 1.236 | **2.789** | 1.093 | **2.774** | 1.085 | **3.457** | 1.224 |
|  | age | **0.869** | 0.199 | **2.160** | 0.679 | **2.195** | 0.719 | **2.428** | 0.781 | **1.928** | 0.686 | **1.913** | 0.675 | **2.437** | 0.777 |
|  | motor2 | **1.484** | 0.737 | **3.897** | 1.799 | **3.933** | 1.851 | **4.384** | 2.008 | **3.436** | 1.728 | **3.400** | 1.713 | **4.396** | 2.017 |
|  | motor3 | **0.319** | 0.630 | **1.557** | 1.310 | **1.571** | 1.332 | **1.777** | 1.433 | **1.328** | 1.243 | **1.304** | 1.231 | **1.785** | 1.431 |
|  | motor4 | **-0.838** | 0.602 | **-1.526** | 1.121 | **-1.553** | 1.128 | **-1.627** | 1.205 | **-1.445** | 1.052 | **-1.434** | 1.062 | **-1.637** | 1.205 |
|  | motor5 | **-1.953** | 0.594 | **-3.685** | 1.277 | **-3.742** | 1.312 | **-4.026** | 1.407 | **-3.406** | 1.237 | **-3.382** | 1.243 | **-4.032** | 1.400 |
|  | motor6 | **-1.473** | 0.986 | **-0.519** | 1.931 | **-0.484** | 1.978 | **-0.158** | 2.143 | **-0.784** | 1.795 | **-0.800** | 1.790 | **-0.161** | 2.131 |
|  | motor9 | **-1.561** | 0.868 | **-0.974** | 1.404 | **-0.969** | 1.415 | **-0.852** | 1.495 | **-1.099** | 1.328 | **-1.107** | 1.333 | **-0.851** | 1.486 |
|  | trial2 | **0.391** | 0.659 | **-0.846** | 1.823 | **-0.900** | 1.883 | **-1.248** | 2.120 | **-0.612** | 1.672 | **-0.600** | 1.650 | **-1.212** | 2.115 |
|  | trial3 | **1.131** | 0.888 | **1.362** | 2.033 | **1.381** | 2.091 | **1.407** | 2.299 | **1.298** | 1.854 | **1.292** | 1.847 | **1.415** | 2.321 |
|  | trial4 | **-0.682** | 0.781 | **-2.034** | 1.860 | **-2.070** | 1.898 | **-2.312** | 2.080 | **-1.809** | 1.722 | **-1.813** | 1.715 | **-2.286** | 2.085 |
|  | trial5 | **0.639** | 0.667 | **1.913** | 1.792 | **1.936** | 1.845 | **2.219** | 2.100 | **1.614** | 1.636 | **1.598** | 1.601 | **2.242** | 2.078 |
|  | trial6 | **1.318** | 0.975 | **2.640** | 2.019 | **2.633** | 2.053 | **2.781** | 2.193 | **2.428** | 1.893 | **2.398** | 1.876 | **2.786** | 2.200 |
|  | trial7 | **1.889** | 0.822 | **1.663** | 1.906 | **1.657** | 1.919 | **1.603** | 2.087 | **1.696** | 1.753 | **1.678** | 1.744 | **1.631** | 2.072 |
|  | trial8 | **0.698** | 0.818 | **-0.469** | 2.338 | **-0.515** | 2.412 | **-0.869** | 2.692 | **-0.242** | 2.142 | **-0.226** | 2.135 | **-0.811** | 2.679 |
|  | trial9 | **2.084** | 1.190 | **4.445** | 3.357 | **4.520** | 3.447 | **4.956** | 3.877 | **3.994** | 3.052 | **3.968** | 3.005 | **4.988** | 3.888 |
|  | trial10 | **0.744** | 0.742 | **0.742** | 1.560 | **0.727** | 1.579 | **0.665** | 1.693 | **0.742** | 1.439 | **0.748** | 1.425 | **0.670** | 1.693 |
|  | trial11 | **0.311** | 0.747 | **0.422** | 1.394 | **0.412** | 1.405 | **0.417** | 1.496 | **0.389** | 1.298 | **0.382** | 1.296 | **0.421** | 1.496 |
